# Development of an integrated peri-urban wetland degradation assessment approach for the Chatra Wetland in eastern India

**DOI:** 10.1038/s41598-021-83512-6

**Published:** 2021-02-24

**Authors:** Tirthankar Basu, Arijit Das, Quoc Bao Pham, Nadhir Al-Ansari, Nguyen Thi Thuy Linh, Gareth Lagerwall

**Affiliations:** 1 Department of Geography, University of Gour Banga, Malda, West Bengal 732103 India; 2 Environmental Quality, Atmospheric Science and Climate Change Research Group, Ton Duc Thang University, Ho Chi Minh City, Vietnam; 3 Faculty of Environment and Labour Safety, Ton Duc Thang University, Ho Chi Minh City, Vietnam; 4 Department of Civil, Environmental and Natural Resources Engineering, Lulea University of Technology, 97187 Luleå, Sweden; 5 Thuyloi University, 175 Tay Son, Dong Da, Hanoi, Vietnam; 6 Bioresources Engineering, School of Engineering, University of KwaZulu-Natal, Scottsville, P. Bag X01, Pietermaritzburg, 3209 Republic of South Africa; 7 The Centre for Water Resources Research, University of KwaZulu-Natal, Scottsville, P. Bag X01, Pietermaritzburg, 3209 Republic of South Africa

**Keywords:** Engineering, Environmental impact

## Abstract

The loss of peri-urban wetlands is a major side effect of urbanization in India in recent days. Timely and proper assessment of wetland area change is essential for the conservation of wetlands. This study follows the integrated way of the peri-urban wetland degradation assessment in the case of medium and small-size urban agglomerations with a special focus on Chatra Wetland. Analysis of land-use and land cover (LULC) maps of the past 28 years shows a decrease of 60% area of the wetland including marshy land. This has reduced the ecosystem services value by about 71.90% over the period 1991–2018. From this end, The Land Change Modeler of IDRISI TerrSet using the combination of MLPNN and Markov Chain has been used to predict the LULC map of this region. The scenario-based modeling following the LULC conversion and nine explanatory variables suggests the complete loss of this wetland by 2045. However, the authors have also tried to present a future LULC pattern of this region based on an environmental perspective. This proposed map suggests possible areas for built-up expansion on the western side of the city without significantly affecting the environment.

## Introduction

Wetlands cover only a marginal area of the earth’s surface i.e. 6.2–7.6%^1^. However, they are considered a vital environmental unit in the world^2, 3^. The Ramsar Convention Secretariat^4^ has recognized the international importance of wetlands as they provide several ecological and environmental benefits in the forms of services and goods like the recharge of groundwater, biodiversity, flood mitigation, food, fisheries, and other economic activities^5, 6^.

However, researchers have observed that rapid urbanization has generated immense stress on the urban/peri-urban wetlands and this situation is most prominent in developing countries. The absence of proper infrastructural plans and planning maps has created very fragmented and chaotic urbanization in most of the developing countries of Asia^7–9^. As a consequence of this, the uncontrolled spillover effect of urbanization has created several adverse effects on ecology and the environment. This has ultimately resulted in a high rate of wetland degradation in these regions^10–14^. The vulnerability of urban/peri-urban wetlands is also expressed in the voice of the executive director of the United Nations Environmental Program, Achim Steiner at the United Nations conference, Hyderabad, India in 2012. He expressed that 50% of wetlands in the world have been damaged during the last 100 years and recommended major land policy reforms for urban/peri-urban wetlands conservation^15^.

The presence of wetlands in fringe areas and their degradation is a common phenomenon of the megacities and large urban agglomerations in developing countries. However, in the South Asian context, the impact of urbanization on wetlands is studied mainly in the case of major metropolitan areas. A few such examples include the degradation of east Kolkata wetlands due to Kolkata urban agglomerations, India^16, 17^, wetland degradation in the vicinity of Dhaka, Bangladesh^18^, and the degradation of urban wetlands of Colombo, Sri Lanka^19^.

Very few records are available for developing a comprehensive impact assessment of urbanization on wetland degradation in non-metropolitan cities for developing countries. Lack of well-documented official data is a major obstacle to the peri-urban wetland degradation assessment in case of small and medium urban agglomerations in India. In this case also, no proper official records are found on the Chatra Wetland and its temporal evolution. This poses limitations in case of assessing dynamic changes through traditional field surveys due to inadequate spatial and temporal sampling^3^. Under these circumstances, geospatial techniques have received wide application throughout the world^20, 21^.

Land use land cover (LULC) change analysis is the most effective way to highlight changes in natural cover^22, 23^. Among the several commonly used models for LULC prediction, this study has adopted the multi-layer perception neural network (MLPNN)-Markov model due to it being included in the Land Change Modeler in the IDRISI TerrSet software^1^. The previous study by Das and Basu^24^ provides a successful application of this software in this region. The spatial characteristics of different LULC types were analyzed with the help of landscape metrics. These are well-established tools that can identify the fragmentation and the degradation of a specific LULC over time^24^.

It is a well-established fact that degradation or loss of natural cover ultimately results in the loss of ecosystem service values (ESVs)^25, 26^. Therefore, the rate of changes in the ESVs over time serves as an important indicator to assess the possible degradation of natural cover^27^. In this regard, the study of Talukdar et al.^28^ in this region successfully established the positive relationship between the loss of wetland area and loss of ESVs. Further, the perception survey study on the Chatra Wetland by Das and Basu^24^ also showed that the loss of ecosystem services suggests the degradation of the wetland.

Previous studies on wetland degradation assessment are mainly associated with either future projection^16, 17^ or ecosystem services loss^29–31^. This makes it quite difficult to assess the wetlands’ vulnerability completely. There have been no comprehensive studies relating the quantitative assessment of Chatra Wetland’s degradation due to urban induced LULC conversions of the wetland area, or future predictions of LULC based on different driving factors of urbanization. E.g. physical, proximity, socioeconomic factors, and neighborhood^32–36^. Further, the assessment of ecosystem service value loss is yet to be made for an urban/peri-urban wetland of non-metropolitan areas. This research gap must be addressed for the sustainable management of urban/peri-urban wetlands of non-metropolitan cities in developing countries. In this study, an integrated method is presented wherein a detailed quantitative assessment of wetland degradation can be performed. The past, present, and future values of the ecosystem services provided by the study area were estimated based on the modified global coefficients given by Costanza^37^. This will enable the representation of the on-going degradation of wetlands more lucidly.

Hence, this study aimed to address the existing research gap by:

1. Assessing the quantitative degradation of wetland areas through LULC change analysis, landscape metrics, and the construction of an urban water index.
2. The prediction of future LULC change in response to urbanization and consequent loss of ecosystem services values for the Chatra Wetland located in the fringe areas of English Bazar Urban Agglomeration far away from Kolkata metropolitan area.

The novel contribution of this study is that it provides a comprehensive detailed structure to assess the degradation of urban/peri-urban wetlands by integrating different quantitative approaches through the application of geospatial techniques.

This study is based on the hypothesis that assessment of quantitative degradation requires an integrated method such as LULC analysis with future projection, estimation of ESVs, and proposing alternative scenarios as part of policy management. Following the hypothesis, this study will address some important questions regarding the sustainable management of Chatra Wetlands. The research question includes—(1) How has the LULC of Chatra Wetland changed over the past 28 years? (2) Is the wetland still in a continuous stretch or has it become fragmented? (3) What will be the future scenario of this wetland? (4) How much ecosystem services value has been lost due to degradation? (5) Is there any alternative way to save this wetland? The prime objective is to assess the quantitative degradation of Chatra Wetland with the help of geospatial techniques by highlighting the spatial–temporal evolution of Chatra Wetland. Lastly, this study has also proposed an alternative LULC plan for urbanization without deteriorating this wetland.

## Materials and methodology

**Figure 1:**
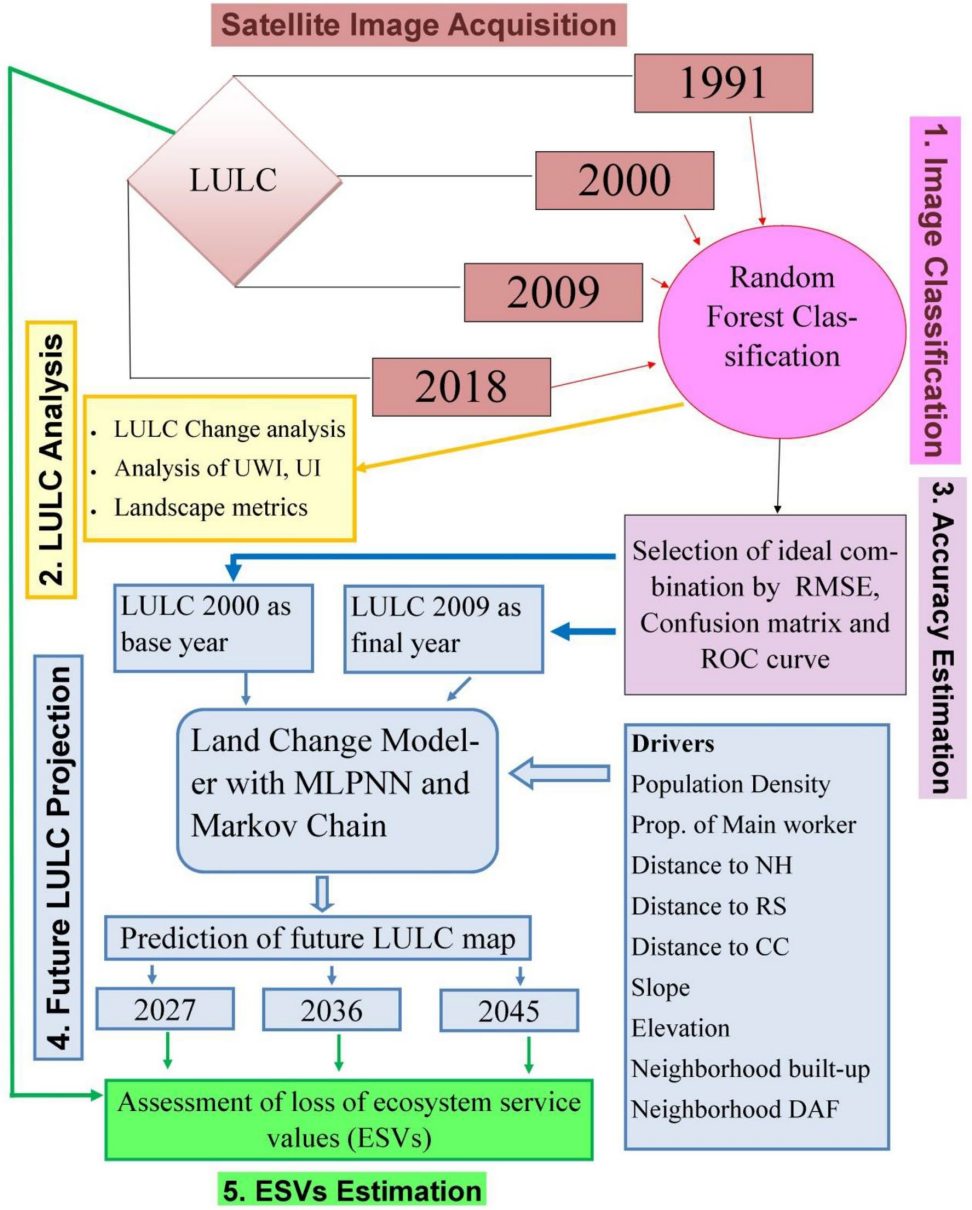
Methodological framework for quantitative assessment of wetland degradation.

The entire methodological section for this purpose is divided into sub-sections to achieve the various objectives. Starting with a brief description of the test site and data collected, the rest of the methodological framework for this section is broken up into five strategies, outlined in Fig. 1, and further elaborated on in Table 1. These strategies represent both the present situation and also a direction to the future.

**Table 1:** Strategies and its application.

Strategy	Method	Purpose
1. Image classification	Image classification by Random Forest (RF) method	LULC map preparation
2. LULC analysis	Urban Water Index (UWI), Urban Land Index (UI), Urban Expansion Index (UI_x_)	To address the shrinking character of Chatra Wetland concerning urbanization
	Landscape metrics	To address the gradual fragmentation of Chatra Wetland
3. Accuracy estimation	RMSE, confusion matrix, ROC	Selection of best LULC model to use for predictive modeling
4. Future LULC projection	Drivers	Spatial data that has an influence on LULC change, as input to the model
	Land Change Modeler	To predict the future LULC of the study area
5. Ecosystem service value (ESV) estimation	ESV	Assessment of environmental degradation (loss of ESV) in the study area

### The site

**Figure 2:**
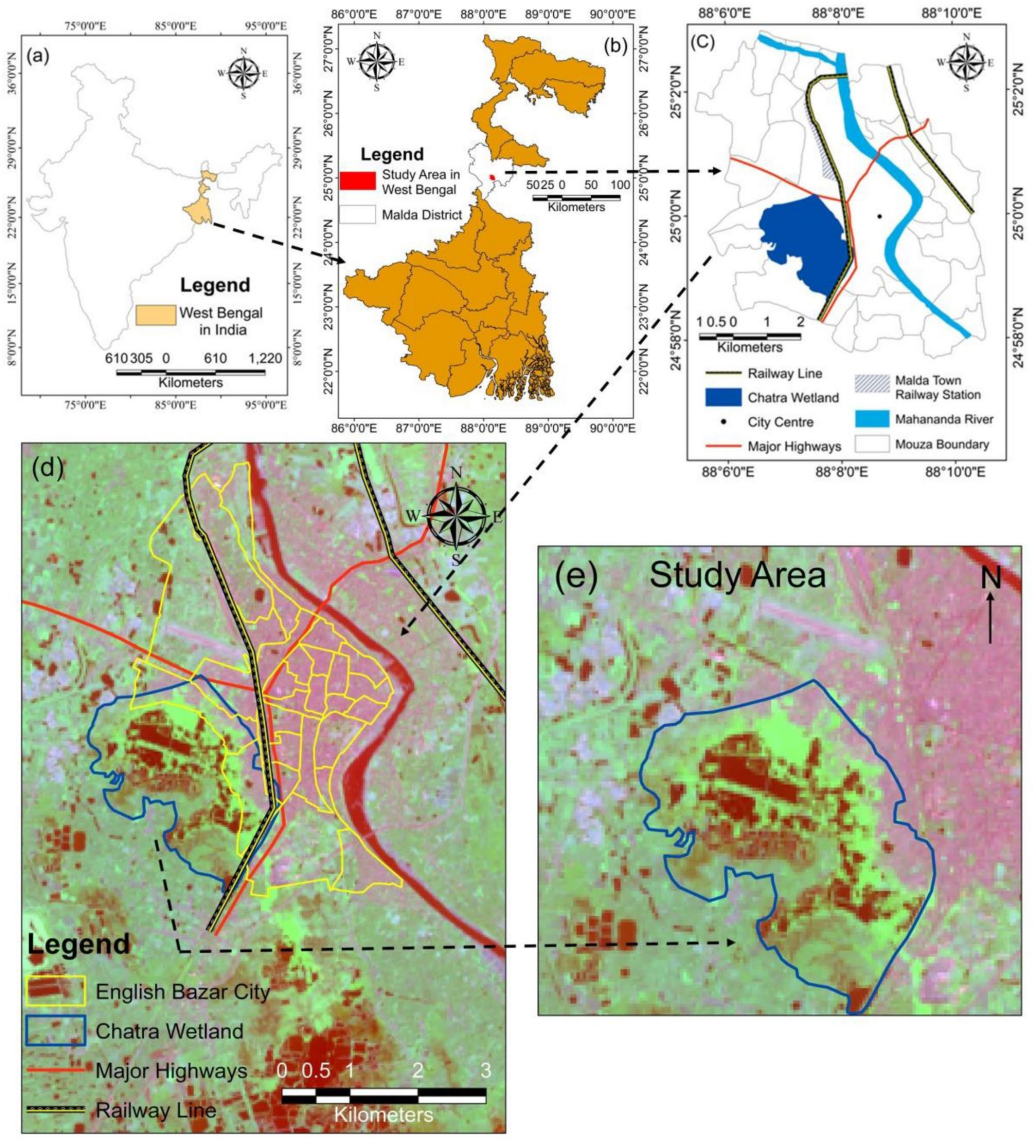
Location of the study area (**a**) West Bengal, the eastern state in India; (**b**) Chatra Wetland in the central part of West Bengal; (**c**) Chatra Wetland with its surrounding areas; (**d**) Location of Chatra Wetland as a peri-urban wetland of English Bazar city; (**e**) Study area taken for this study^46^.

English Bazar City (Fig. 2) is an important city of West Bengal, which is situated around 327 km away from Kolkata (the capital of West Bengal) and in the middle of West Bengal, India. It has a long colonial history^38^ and currently, it serves as Malda District’s headquarters. Shaw and Das^39^ have noticed that the city has expanded around 30% in the last 28 years and around 3,13,681 persons reside in this urban agglomeration^40^.

The Chatra Wetland (Fig. 2), situated in the English Bazar city, is an important region known as the ‘kidney of the English Bazar’^41^. It is mainly a rain-fed wetland, but due to its vast extent, water remains in it throughout the year. The site is located on the plains region of Gangetic plain with elevation ranges from 37 to 7 m. This region is made with the newer alluvium of Ganga and Mahananda, which is known as ‘Diara’. It supports good drainage and fertile soil which is also a reason for the high population density in this region^42^. Chatra Wetland provides a variety of ecosystem services to the city dwellers of English Bazar urban agglomeration such as recharging of groundwater, cooling effect, flood control, biodiversity, food, recreation, etc. On-field observation has revealed that the people of adjacent areas of this wetland are directly dependent on it for fishing, drinking water, agriculture, etc. This wetland is very rich with diversified living organisms.

Delineation of the actual boundary of this wetland is a very difficult task due to its rain-fed character, so this study has followed past articles^41^ to demarcate the boundary of this wetland. It stretches over an area of 8.47 km^2^. Unlike other wetlands, this wetland consists of the marshy tract and the areas with visual surface water. Marshy tracts were demarcated with the help of on-field observation and Google Earth Imageries. LULC is prepared accordingly to compute ecosystem service values (ESVs) separately. However, rapid urbanization has resulted in shrinkage of the diameter of this urban wetland^41, 42–45^.

### Data

This study predominantly made use of secondary data. Secondary data is mainly used for the quantitative assessment of Chatra Wetland degradation. These data have been collected from various sources and detail regarding this is represented by Table 2.

**Table 2:** Data source and its application.

Types of data	Year	Source	Purpose
Landsat TM/OLI (resolution 30 m) (Path: 139; Row: 43)^a^	1991, 2000, 2009, 2018 (Acquired for the MONTH of November)	USGS Earth Explorer	LULC map
Population enumeration Data	2011	Census of India	Population density and percentage of main workers to total workers map generation
Topographic data like slope, elevation	2014	SRTM DEM (30 m resolution) downloaded from USGS earth explorer	Slope map and elevation map
Associated other data like the location of roads, city centre, Railway station	2018	Google earth	Prediction of LULC

^a^Detail description of the Landsat images of the study area is provided in the supplementary section.

### Image classification

Classification of satellite image helps to extract and visualize thematic information in a better way than a raw satellite image. It involves the allocation of spectral signatures for various classes i.e. LULC categories^47^. Spatial–temporal LULC map helps to detect the changes in the area of different LULC^48^ by highlighting the trend and pattern of LULC change of a particular place. Recently, researchers have developed many methods to classify the satellite image^49–51^. Some popular methods of image classification are the maximum likelihood method, random forest (RF), support vector machine (SVM), etc.^51–53^.

This study has applied all three methods individually to classify the satellite image of the study area for the years 1991, 2000, 2009, and 2018. In this regard, 75 sample signatures for each LULC were collected separately for each satellite image in ArcGIS 10.2.1^46^. Six LULC categories were identified in the LULC map of the study area. These are built-up areas, vegetation, marshy land, river, wetland, and DAF (Deforested/Agriculture/Fallow) Land. The LULC classification schematic is represented in Table 3. Finally, maximum likelihood method of image classification was performed in ArcGIS 10.2.1, and image classification through RF and SVM was executed using the R statistical software^54^.

**Table 3:** Land Use and Land Cover classification schematic.

LULC category	Characteristics	Description
Builtup area	Consists of	All kinds of built-up areas including human settlement
	Significance	Represents areas with high population pressure
Vegetation	Consists of	Natural vegetation, suburbs, Grasses
	Significance	Represents green spaces of the study area
Marshy land	Consists of	Swamp
	Significance	Represents the extent Chatra Wetland and Urban water habitat
River	Consists of	The active part of River
	Significance	Represents potable/non-potable and irrigation water source
Wetland	Consists of	Areas of wetland with visual surface water cover
	Significance	Represents the extent Chatra Wetland and Urban water habitat
DAF land	Consists of	Devegetated land, Agricultural land, Fallow land
	Significance	Highlights the areas of encroachment

Accuracy of each classified image was measured by a confusion matrix^55, 56^ based on 278 selected points (separately for each classified year) from toposheet of Survey of India and Google Earth Images. Samples were collected using stratified random sampling and the sample size was determined following Cochran^57^. Overall accuracy of each classified image is shown in Table 4. This study has adopted the output of an RF image classifier for further analysis due to having better accuracy over others.

**Table 4:** Accuracy assessment of Land Use and Land Cover (LULC) by the confusion matrix.

Year	1991	2000	2009	2018
Confusion matrix value for maximum likelihood	0.85	0.86	0.87	0.85
Confusion matrix value for RF	0.87	0.88	0.89	0.88
Confusion matrix value for SVM	0.84	0.83	0.85	0.84

### LULC analysis

The following two subsections discuss various indicators and metrics that were used to evaluate the historical LULC change of Chatra Wetland.

#### Urban Water Index (UWI), Urban Land Index (UI), and Urban Expansion Index (UI_x_)

Normalized Difference Water Index (NDWI) is a popular tool to acquire the area of the water body from satellite images. However, for this study, one particular index is required that can relate the changes in the water area to changes in land use. In this regard, the Urban Water Index (UWI) has been devised following Haas et al.^58^ (Eq. 1). This index helps to quantify the development of Chatra Wetland as compared to simultaneous urban development. Two more indexes (Eqs. 2, 3) have been used to represent the urban development within the area of Chatra Wetland. These are the Urban Land Index (UI) and Urban Expansion Index (UI_x_)^58^. UI has been defined as the ratio between existing urban land and total land. The UI_x_ depicts the changes in urban land as devised by Hu et al.^59^. In this study, the negative effect of urbanization on the Chatra Wetland was assessed by using these three indices i.e. UI, UI_x,_ and UWI.

1 $$UWI = \frac{{AW_{t2} - AW_{t1} }}{{(AUB_{t2} + ADAF_{t2} ) - (AUB_{t1} + ADAF_{t1} )}}$$

2 $$UI = \frac{AUB}{{TL}} \times 100\%$$

3 $$UI_{X} = \frac{{AUB_{t2} - AUB_{t1} }}{{AUB_{t1} }} \times 100\%$$

where UWI, Urban water index; AW_t1_, Area of water body in the initial year; AW_t2_, Area of water body in the final year; AUB_t1_, Area of urban built-up in the initial year; AUB_t2_, Area of urban built-up in the final year; AUB, Area of built-up; TL, Total land area; ADAF_t2_, Area of DAF land in final year; ADAF_t1_, Area of DAF land in the initial year.

#### Landscape metrics

Landscape metrics are successfully applied by numerous studies in case of analysis of landscape patterns of wetland^34, 60, 61^. It helps to investigate the spatial distribution pattern of different LULC at the patch level^62, 63^. In this way, it can highlight the fragmentation and connectedness among the patches of a particular landscape. A gradual decrease in the connectedness over time suggests possible fragmentation and degradation of that particular LULC^24^. In this study, five landscape metrics were selected following McGarigal and Marks^64^ to highlight the landscape fragmentation of Chatra Wetland. The details on these metrics are provided in Table 5. All the landscape metrics were applied using Fragstats v4.2.1^64^ software against each classified image of Chatra Wetland.

**Table 5:** Selected landscape metrics and their description.

	Description
**Landscape composition metrics**	
Patch density (PD)	Depicts the number of patches in every km^2^
Area-weighted mean patch size (AMPS)	Depicts average area-weighted patch size of each class
Largest patch index (LPI)	Represents the percentage of area occupied by the largest patch of a class
**Landscape configuration metrics**	
Cohesion	Depicts connectedness among the patches of the respective class. A higher value indicates high aggregation among the patches
Contagion (CONTAG)	It is a measure of relative aggregation among the patches at the landscape level. Value ranges from ‘0’ to ‘100’. ‘0’ represents highly disaggregation and ‘100’ represents highly aggregation

### Accuracy estimation

In this study, two MLPNN classification models were tested—one using the 1991–2000 LULC combinations and the other using the 2000–2009 LULC combinations. The best performing model would then be used in predicting the future scenarios. More information relating to the development of the models themselves is found in “Land Change Modeler” section below.

The models were validated by using the root-mean-square-error (RMSE), confusion matrix, and receiver operating characteristics (ROC) curve. The RMSE is well known for determining error of predictions, and the confusion matrix was discussed previously in the image classification section. The ROC is an estimator of goodness of fit of the model, with a value of 0.5 indicating complete randomness, and a value of 1 indicating an exact model without error^65, 66^. As per the recommendation of Olofsson^67^, the reference map of validation should be more accurate than the classification map. Therefore, the 1991–2000 LULC model was used to predict a 2009 map, and the 2000–2009 LULC model was used to predict a 2018 map. The validation was performed by comparing the predicted 2009 and 2018 LULC maps with the respective satellite imagery of the area of interest (Acquired from the Google Earth Pro version).

In this regard, 300 points (pixels) were selected randomly to carry out the validation by RMSE, confusion matrix, and ROC curve. A good video tutorial about the implementation of the confusion matrix in R-Studio statistical software is available on YouTube^68^ and the ROC curve is prepared in SPSS statistical software 25.0^69^. RMSE was estimated following Eq. (4)^70^.

4 $$RMSE = \sqrt {\frac{{\sum\nolimits_{i = 1}^{N} {(\hat{I}_{i} - I_{i} )^{2} } }}{N}}$$

where $\hat{I}_{i}$ is the predicted value for sample i; $I_{i}$ is the observed value of sample i; N represents the total number of observations (samples).

### Future LULC projection

The following subsections relate to determining future scenarios of Chatra Wetland and include explanatory variables for the transition potential; transition potential map calculation and simulation; and accuracy assessment for determination of the ideal combination of variables. These future scenarios were predicted using the best model as determined from the previous subsection (i.e. either the 1991–2000 LULC combination or the 2000–2009 LULC combination).

#### Drivers

Prediction of future Land Use and Land Cover (LULC) enables the researchers to foresee the future LULC pattern and helps to estimate the demand for land use in the future^71^. Furthermore, it provides a guide to take up effective policies for the effective management of natural resources. The selection of a proper set of input variables is essential to produce optimum future projection. Previous studies have pointed out a few influential groups of drivers behind LULC change i.e. physical, Proximity, socio-economic factors, neighborhood factors, etc.^32–36, 72–74^. However, there are no definite universal explanatory variables in this regard.

The dynamics of urban growth and peri-urban growth in this region was analyzed by Dutta and Das^75^ and Shaw and Das^39^. From these findings, it can be concluded that driving factors like accessibility to public services, economic opportunity, political influence, and population growth, are the main driving forces behind the existing LULC transformation in this region. However, it is essential to mention that some of the driving variables (like political situation) are of non-spatial characteristics and it is difficult to incorporate in the prediction model. Besides this, formal consultation with the planning expert of English Bazar Municipality and careful analysis of four LULC transformation stages (1991–2000, 2000–2009, and 2009–2018) have identified a set of driving variables that have provided stimulus behind the LULC transformation in the study area.

Finally, nine driving variables having spatial characteristics were taken into consideration for this study. These factors are: the distance from National Highways, distance from the railway station (Malda Town railway station), distance from the city centre, population density, and proportion of main worker (workers engaged in work for more than 180 days in a year) in different mouzas (villages) as per census 2011, elevation, slope, neighborhood built-up, and DAF cells in LULC. The details of these variables are presented in Table 7. The raster layers of these variables have been generated in ArcGIS 10.2.1.

It is important to mention that all the parameters do not influence the rate of urbanization in the same way. For example, a high population density of a region will create more pressure for the conversion of natural resources. Contrary to this, the rate of urbanization will be high in those areas which lie near the city centre. Therefore, a standardization procedure was followed to remove the contrasting character of the dataset and to make all the layers unidirectional with an equal scale. Some of the popular standardization techniques are 10 point scale^76^, Analytic Hierarchy Process (AHP)^77^, fuzzy standardization^17^, etc.

The main advantage of fuzzy standardisation membership functions over other methods is that it can work in an uncertain system where theory seems inadequate but expert judgment alone can make sense^78, 79^. Fuzzy membership transforms all the layers in a continuous crisp membership form which ranges from 0 to 1. The value of ‘1’ denotes membership of the set and ‘0’ denotes non-membership. Following McBratney and Odeh^80^, a sample of the fuzzy set can be represented as Eq. (5).

5 $$A = \{ x,\mu_{A} (x)\} \,{\text{for}}\,{\text{each}}\,x\varepsilon X$$

where $\mu_{A}$ = membership of x in the fuzzy set A; $\mu_{A}$ = 0 if x does not belong to the set A; $\mu_{A}$ = 1 if x totally belongs to the set A; $\mu_{A}$ = 0 < $\mu_{A}$(x) < 1 if x belongs to a certain degree in set A.

In this study, fuzzy sigmoid membership functions like ‘Large’ or ‘Small’ or ‘MS Large’ have been applied to driving factors (Figure S1. See supplementary section). This step was performed to make all the driving factors unidirectional and to give all the factors an equal comparable scale^17^. A description regarding these functions is provided in Table 6 and the allocated fuzzy membership and logic are shown in Table 7. After fuzzy standardization, these variables were imported into the IDRISI environment^1^.

**Table 6:** Description of Fuzzy membership functions.

Membership function	Description	Definition
Large	Sigmoid shape with larger values having larger membership^81^	$\mu (x) = \frac{1}{{1 + \left( {\frac{x}{{f_{2} }}} \right)^{{ - f_{1} }} }}$ where f_1_ = spread of membership value from 0 to 1; f_2_ = mid-point having membership value as 0.5
Small	Sigmoid shape with small values having larger memberships^81^	$\mu (x) = \frac{1}{{1 + \left( {\frac{x}{{f_{2} }}} \right)^{{f_{1} }} }}$ where f_1_ = spread of membership value from 0 to 1; f_2_ = mid-point having membership value as 0.5
MS large	Sigmoid shape wherein mid-point and spread of membership values are defined by the mean and standard deviation of the data. Here, larger values represent larger membership^82^	$$\begin{gathered} \mu (x) = 1 - \frac{bs}{{x - am + bs}}if,x > am,Otherwise \hfill \\ \mu (x) = 0 \hfill \\ \end{gathered}$$where m represents mean; s represents standard deviation; a and b are the multiplier

**Table 7:** Description of selected variables and Fuzzy standardization process.

Category	Variable	Description	Fuzzy standardization	
			Membership function	The reason behind the allocation of membership
Socio-economic	Population density	The map is generated at mouza (village) level for the year 2001 and 2011 by taking information from the Census of India	Large	High population pressure will result in the expansion of built-up and DAF land^83^
	Proportion of marginal workers to total workers		Large	A greater proportion of main workers suggests relatively more economic opportunity and will attract more people that will ultimately create more pressure on natural resources^84^
Physical factor	Slope	Slope map (30 m resolution) is generated in degree format using ‘slope’ spatial analyst tool in ArcGIS 10.2.1	Large	This Region is almost flat. Still, the areas with slightly higher elevation and the slope will act as drypoints^71^
	Elevation	Digital Elevation Model with 30 m resolution is extracted for the study area from USGS earth explorer	Large	
Proximity factor	Distance from National Highway (NH)	Location of National Highway, railway station and city centre within the study area has been digitized from Google earth. Then, the Euclidian distance map is generated for each variable	Small	Rate of conversion will be high nearby^41^
	Distance from the railway station (RS)		Small	
	Distance from the city centre (CC)		Small	
Neighbourhood factor	Proportion of built-up land in the surrounding region	The map is generated using ‘Block Statistics’ spatial analyst tool in ArcGIS 10.2.1 having 2 × 2 neighborhood cells	MsLarge	Neighborhood built-up or DAF cell will create the possibility of conversion of that cell into built-up or DAF^85^
	Proportion of DAF land in the surrounding region		MsLarge	

#### Land Change Modeler

Prediction of future LULC includes the creation of a transition potential map to represent the scenarios of future LULC. However, it is important to mention that English Bazar is not a planned city and there is no proposed LULC plan for this city in the upcoming 25 years. So, it is quite expected that the expansion of this city will occur in the same way in the near future as it is today. Hence, scenarios based on “as usual growth” was taken into consideration for the projection of the condition of Chatra Wetland in the future following the work of Thapa and Murayam^71^. The entire operation of future LULC prediction was performed by the IDRISI TerrSet software^1^ with the help of its Land Change Modeler (LCM) extension. The simulation procedure in Land Change Modeler has been designed in a combination of Multi-Layer Perception Neural Network (MLPNN) and Markov framework. MLPNN^86^ classifiers can be described by Eqs. (6) and (7)^70^.

6 $$net_{j} = \sum\limits_{{}} {w_{ij} I_{i} }$$

where net_j_ represents the input that a single node j receives; W_ij_ refers to the weights between node i and j; I_i_ represents the output of node i of an input or hidden layer.

Furthermore, output (O_j_) from the j node was calculated as follows:

7 $$O_{j} = f(net_{j} )$$

where function ‘f’ represents a non-linear sigmoidal function.

The main advantage of MLPNN is that no prior assumptions are required while modeling^87–89^. MLPNN performs the integration of all the explanatory variables, whereas, Markov model determines the transition potential in the case of each LULC type. In this study, transition potential maps were generated following two different combinations. First, The LULC map of 1991 was selected as the base year and 2000 was selected as a final year to predict the LULC map of 2009. After that, the base year was replaced as the LULC map of 2000, and the final year was selected as the 2009 LULC map to predict the LULC map of 2018. For the prediction, the number of samples was selected following the guidelines of Olofsson et al.^67^ using stratified random sampling. Equation (8) was used following Cochran^57^ to generate the overall sample size. Equation (9) was used to determine the number of hidden layers.

8 $$n = \frac{{(\sum {W_{1} S_{1} } )^{2} }}{{[S(\overset{\lower0.5em\hbox{$\smash{\scriptscriptstyle\frown}$}}{O} )]^{2} + (1/N)\sum {W_{i} S_{i}^{2} } }} \approx \left(\frac{{\sum {W_{i} S_{i} } }}{{S(\overset{\lower0.5em\hbox{$\smash{\scriptscriptstyle\frown}$}}{O} )}}\right)^{2}$$

where N represents the number of units (pixels) in the area of interest; W_i_ represents the mapped proportion of class I; S_i_ represents the standard deviation of stratum I and S($$\overset{\lower0.5em\hbox{$\smash{\scriptscriptstyle\frown}$}}{O}$$) represents the standard error of the estimated overall accuracy which is likely to achieve.

9 $$N_{h} = INT\sqrt {N_{i} \times N_{o} }$$

where N_h_ represents the number of hidden layer nodes; h represents the hidden layer; N_i_ represents the number of input layer nodes; i represents the input layer; N_o_ represents the number of output layer nodes; o represents the output layer.

Finally, all the nine driving variables were incorporated in the data sub-model structure and the transition potential maps were generated by MLPNN with a 1025 sample size. Equation (9) suggests that the number of hidden layers will be six. However, after several cross-validations, 5 hidden layers were selected to run the model due to better output (results). In LCM, MLPNN automatically performs the training process by generating training cells from the transition of land for each LULC type. It creates a multivariate function that helps to predict the transition probability of any cell based on the values of explanatory variables. In LCM, more than 80% of classification accuracy is generally recognized as good to carry out the prediction analysis^71, 90, 91^. In this study, the MLPNN model has displayed the classification accuracy rate of 84.20% and 86.42% after 10,000 iterations for the first and second combinations respectively.

### ***ESVs ***estimation

The term ecosystem services refer to all the material and non-material benefits that the living organisms enjoy from a particular environmental unit^92^. Compact natural cover with no significant loss in natural cover will deliver steady ESVs over time. Previous studies showed that the conversion of the natural cover into anthropogenic land uses led to negative changes in the overall ecosystem service values (ESVs)^28, 93^.

The pattern of changes (positive/negative) in the ESVs helps to understand the loss of natural cover and helps to undertake necessary strategies for its management. Therefore, temporal estimation of ecosystem service value serves an important role to assess the rate of degradation of the environment^73, 94, 95^. To show the impact of urban growth on the ecosystem, calculation of ecosystem service value of each LULC (Waterbody, Vegetation, Urban, Agricultural land, sand bar, etc.) was necessary^29^.

Costanza et al*.*^92^ issued a list of coefficient values for different LULC to estimate the ecosystem service values based on a simple benefit transfer method. However, subsequent assessments based on a larger number of case studies have updated the ESVs estimation^37^. Modification in the value of coefficients has been done based on the report of de Groot et al.^96^. This report consists of aggregated values of 22 ecosystem services, which were prepared based on 665 value estimations from over 300 case studies in different parts of the world. This study used the modified global coefficient values of ESVs estimation following Costanza^37^. A previous study by Das and Das^29^, showed the successful application of these modified coefficient values to estimate the ESVs in the tropical areas of West Bengal.

Following this, the ecosystem service values were calculated using Eqs. (11) and (12).

11 $$ESV = \sum {(A_{k} \times VC_{k} )}$$

12 $$ESV_{f} = \sum {(A_{k} \times VC_{fk} )}$$

where ESV = Ecosystem service value of a particular year; A_k_ = Area in hectare; VC_k_ = Value coefficient ($/ha/year) for land class ‘k’; ESV_f_ = Ecosystem service value of function ‘f’; VC_fk_ = Value coefficient for function ‘f’ ($/ha/year) for land class ‘k’.

## Results and analysis

The following results are covered in two sections, those relating to the historical degradation of Chatra Wetland, and those relating to future scenarios and ecosystem services.

### The quantitative degradation of Chatra Wetland

This section is further subdivided into consideration of LULC changes, UI, UIx, and UWI changes, and an analysis of landscape metrics.

#### Analysis of LULC changes from 1991 to 2018

**Figure 3:**
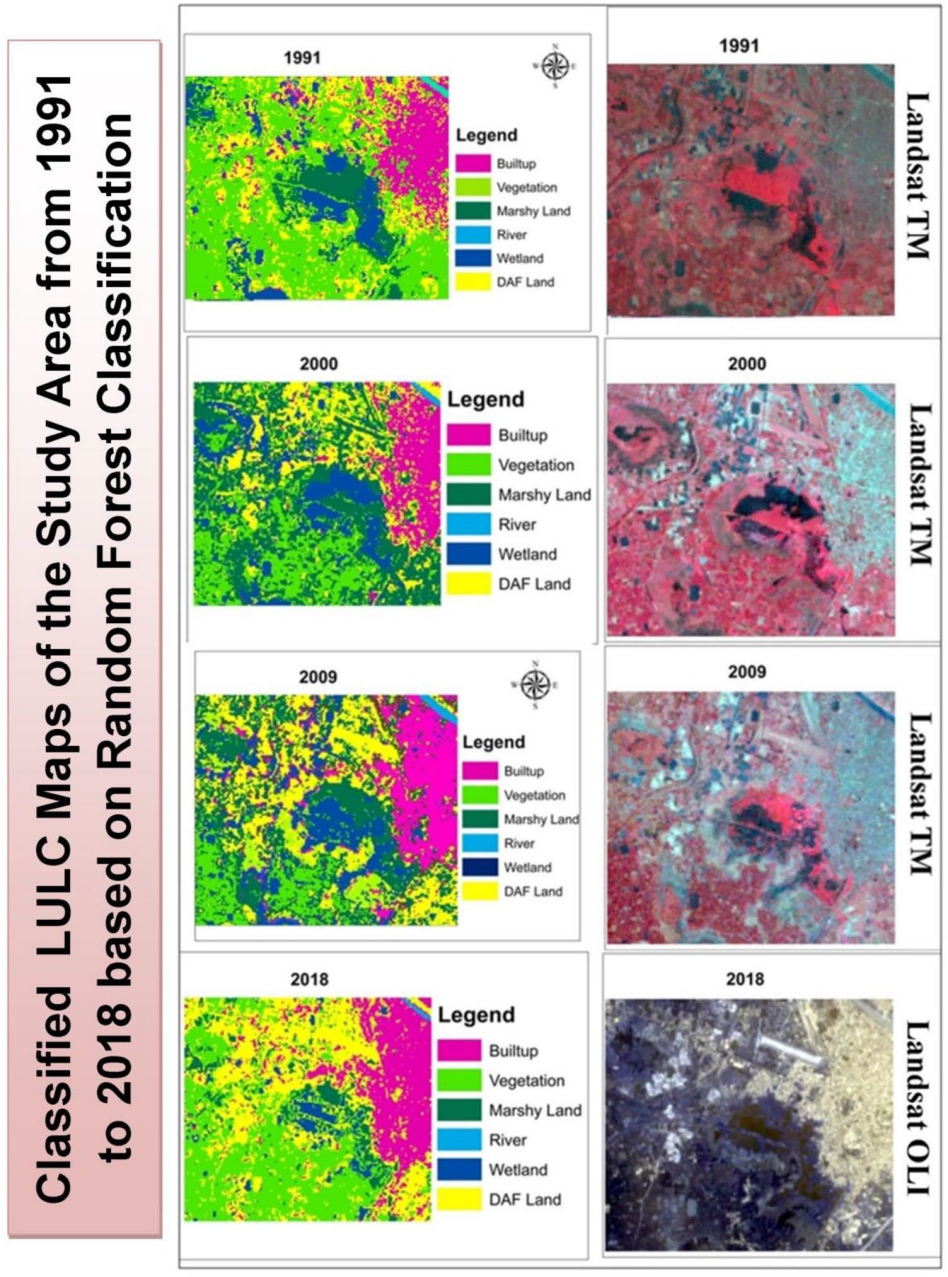
Classified Land Use and Land Cover of Chatra Wetland and its Surrounding for the year (**a**) 1991; (**b**) 2000; (**c**) 2009; (**d**) 2018^46, 54^.

Analysis of LULC (1991–2018) of the study area shows a gradual decrease in the net area of Chatra Wetland and it represents the transformation of Chatra Wetland from a continuous stretch to a discontinuous stretch (Fig. 3). In 1991, around 660.6 hectares was under the marshy land and wetland in the study area. However, the classified LULC map of 2000 (Fig. 3) displays a significant increase in the area of Chatra Wetland and a registered 1089.18 hectares under the wetland and swamp area. This was due to devastating flooding in English Bazar city and its surrounding region in 1998 and 1999^97^. The vegetated areas were converted into swamp areas due to waterlogging at this time^98^. This was the only exception year that shows an increase in the net area of marshy land in the study area. After nine years (2000–2009), the area under wetland and swamp has reduced significantly (Table 8) and it came down to 280.98 hectares with a decrease of about 74.28%.

**Table 8:** Land Use and Land Cover (LULC) area of Chatra Wetland and its surrounding from 1991 to 2018.

LULC category	Year				Net increase or decrease of the area in hectare from 1991 to 2018	Equivalent LULC for ESV
	1991	2000	2009	2018		
	Area in hectare	Area in hectare	Area in hectare	Area in hectare		
Built-up	457.2	546.66	568.35	570.29	+ 113.09	Urban
Vegetation	1023.75	465.12	1030.59	810.54	− 213.21	Tropical forest
Marshy land	263.43	773.28	178.38	161.01	− 102.42	Swamp
River	15.81	14.11	13.5	13.5	− 2.31	Rivers/lakes
Wetland	397.17	315.9	102.6	101.66	− 295.51	Wetland
DAF land	231.34	272.81	494.46	730.88	+ 499.54	Cropland

Contrary to this, the built-up area and DAF land have increased from 819.47 hectares to 1062.81 hectares with an increase of about 29.69%. A significant increase has also been observed in the case of the vegetated area as well. This situation has come about due to the growth of small trees and grasses in place of swamp or wetland areas. As a result, in 2009, the vegetated area has increased by about 121.57% (from 465.12 hectares in 2000 to 1030.56 hectares in 2009). This time vegetal cover was the main net contributor to the area lost by wetland and swamp area during this time frame (2000–2009).

However, the situation has changed drastically in 2018. A large portion of vegetal cover has been removed from this region and converted into DAF and built-up land for commercial purposes. Some portion of wetland and swamp area in the eastern side of English Bazar was further encroached and infilled for residential purposes. As a result, the expanse of wetland and swamp area has further reduced down between 2009 and 2018 and around 6.52% of the area has been converted into other land use. In 2018, the area under vegetated land came down to 810.54 hectares, while built-up and DAF land at the same time has registered an area of about 1301.17 hectares with an increase of 22.43%.

However, it is mentioned earlier that Chatra Wetland is mainly a rain-fed wetland. Therefore, analysis of rainfall trend from 1991 to 2018 is essential to find out the role of rainfall in the shrinking character of Chatra Wetland. In this regard, one existing study^99^ showed that there was no significant deviation in the rainfall trend of the Malda District during this period. Therefore, the period of 2000–2018 has registered a significant deterioration of environmental units in this region due to mainly the LULC conversion, and the Chatra Wetland has suffered the most.

#### UI, UI_x_, and UWI changes

**Figure 4:**
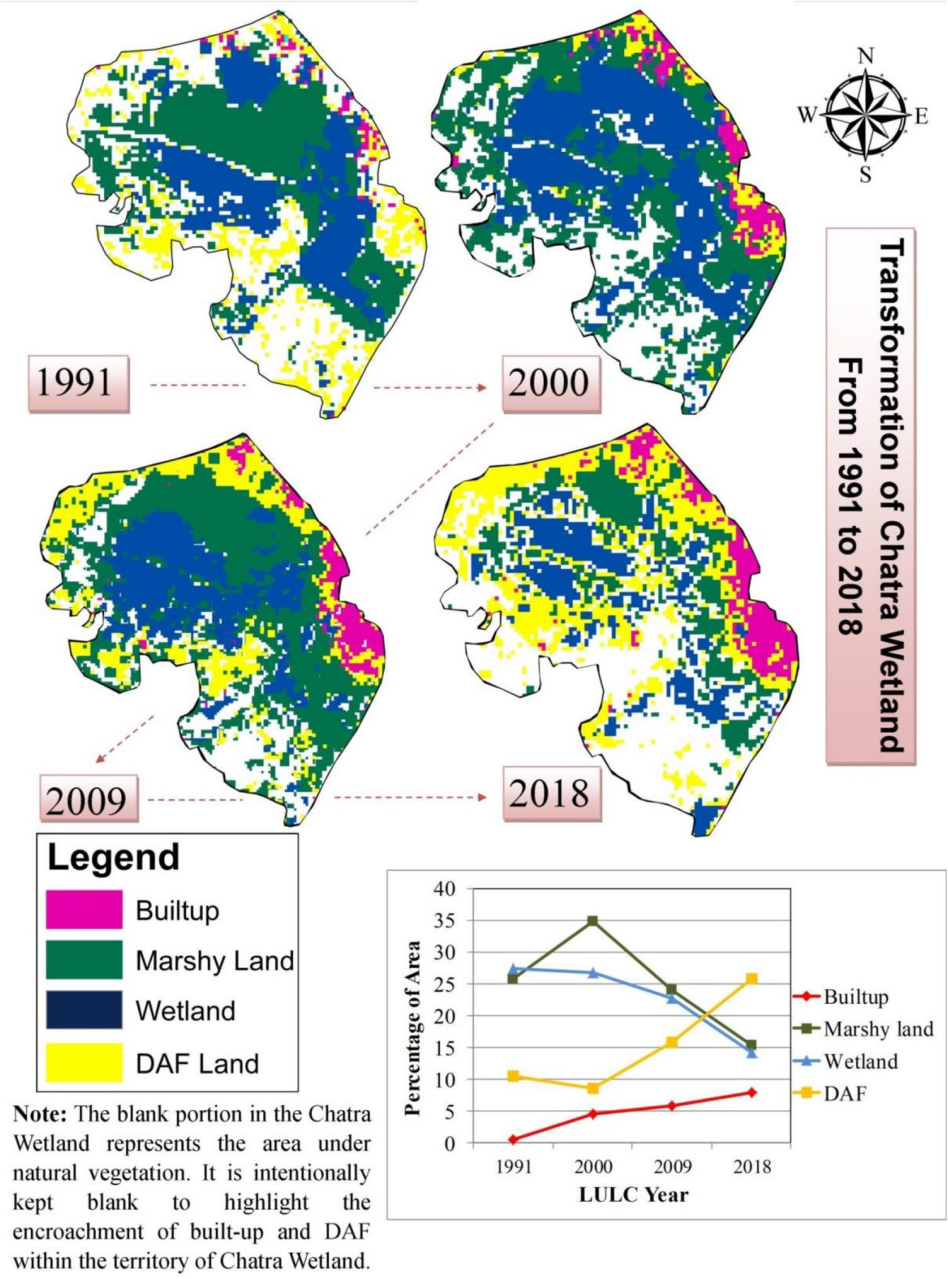
Land Use and Land Cover (LULC) pattern within the area of Chatra Wetland^1^.

The application of these indices was carried out only in case of the area of Chatra Wetland (Fig. 4) to understand the encroachment by urbanization. UI shows that the presence of urban land was only 0.52% (0.027 km^2^) in 1991 within the demarcated area of Chatra Wetland. However, this value has increased to 4.49% (0.23 km^2^) in 2000 despite the overall increase of marshy land in the study area. UI_x_ indicates a 765.56% increase in urban land within the boundary of Chatra land at this time.

However, the share of DAF land has decreased by 1.94% (around 0.1 km^2^). UWI also indicates an increase (4.108%) (Table 9) in wetland cover. Few portions of DAF land emerged out as a marshy land in 2000. However, the share of wetland with visual surface water cover has decreased from 27.42 to 26.74% during 1991–2000. A positive increase in UWI was the result of an increase in marshy land due to the waterlogging event. Encroachment in Chatra Wetland by urbanization has further increased during the subsequent periods.

**Table 9:** Output of urban water index (UWI), urban land index (UI), and urban expansion index (UI_x_).

Indices	LULC year			
	1991	2000	2009	2018
*UWI*		4.108	− 1.763	− 1.391
*UI*	0.519	4.491	5.763	7.901
*Uix*		765.556	28.327	37.079

However, the next phase (2000–2009) has registered the lowest rate of urban expansion within the periphery of Chatra Wetland. The urban land has increased by 28.32% and its share has reached 5.76% in 2009. This time DAF land has remained the main force behind the shrinkage of the wetland area with a 15.78% share. Consequently, a significant decline has been observed in UWI (− 1.76% as compared to urban land and DAF) and the share of wetland with visual surface water and marshy land has been reduced to 22.75% and 26.74% respectively (Fig. 4).

The phase of 2009–2018 again registered an increasing urbanization effect on the wetland. The proportion of urban land and DAF land has increased to 7.90% and 25.89% respectively. The rate of urban land increase (UI_x_) during this time was about 37.08%. As against this, the share of the wetland has reduced by 1.39% and only 29.70% area with the recognized Chatra Wetland area has remained as a true wetland.

Overall, urbanization has caused 1.02% shrinkage in the area of Chatra Wetland from 1991 to 2018. Besides this, assessment of the relationship between the loss of Chatra Wetland and the expansion of agricultural land has also revealed a significant role (Pearson’s correlation value 0.92; R^2^ = 0.66 with *p* < 0.001) of this factor behind the destruction of this wetland in the western margin.

#### Analysis of landscape metrics

Analysis of the past 28 years’ (1991–2018) LULC has revealed significant shrinkage in the area of Chatra Wetland. The LULC of the study area in 1991 shows an almost continuous stretch of Chatra Wetland. However, the visual representation of subsequent LULC shows fragmentation in the landscape of Chatra Wetland. This section deals with the identification of possible landscape fragmentation of Chatra Wetland by using five landscape metrics.

The decreasing percentage of the landscape of Chatra Wetland with an increase in patch density (PD) (Table 10) indicates a greater number of small patches in a comparatively lesser area. It also confirms the shrinkage of Chatra Wetland over time. The declining trend of LPI and AMPS suggests disaggregation among the patches of Chatra Wetland as the larger patches are disintegrating into smaller patches. This fact is supported by a relative decrease in the connectedness among patches as the degree of cohesion among the patches has decreased from 94.54% in 1991 to 87.71% in 2018. The relatively lower value of CONTAG also indicates small and dispersed patches of wetland.

**Table 10:** Results of different landscape metrics for Chatra Wetland.

LULC year	PD	LPI	AMPS	CONTAG	COHESION
1991	36.921	37.210	59.883	51.159	94.731
2000	37.797	27.325	36.788	47.606	94.539
2009	69.889	20.186	24.011	46.239	92.647
2018	71.479	14.036	9.126	34.827	87.706

### Future scenarios and ecosystem services of Chatra Wetland

This section is further divided into an analysis of the future scenarios, and an assessment of the loss of ecosystem services.

#### Analysis of the future scenarios of Chatra Wetland

**Figure 5:**
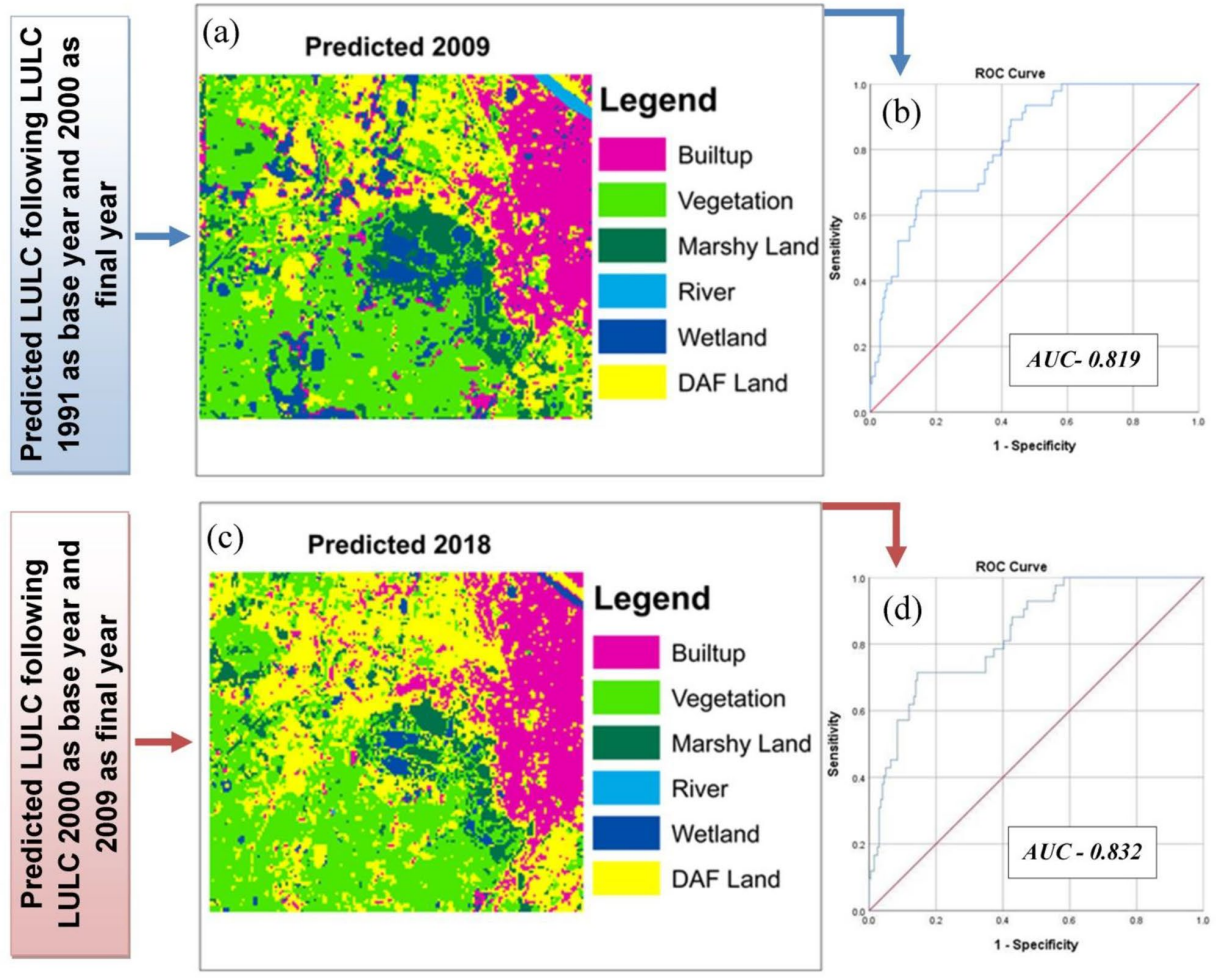
Validation of predicted Land Use and Land Cover (LULC) (**a**) predicted LULC 2009 by taking the LULC of 1991 and 2000; (**b**) ROC curve for 2009 predicted LULC; (**c**) Predicted LULC 2018 by taking LULC of 2000 and 2009; (**d**) ROC curve for 2018 predicted LULC^1^.

**Figure 6:**
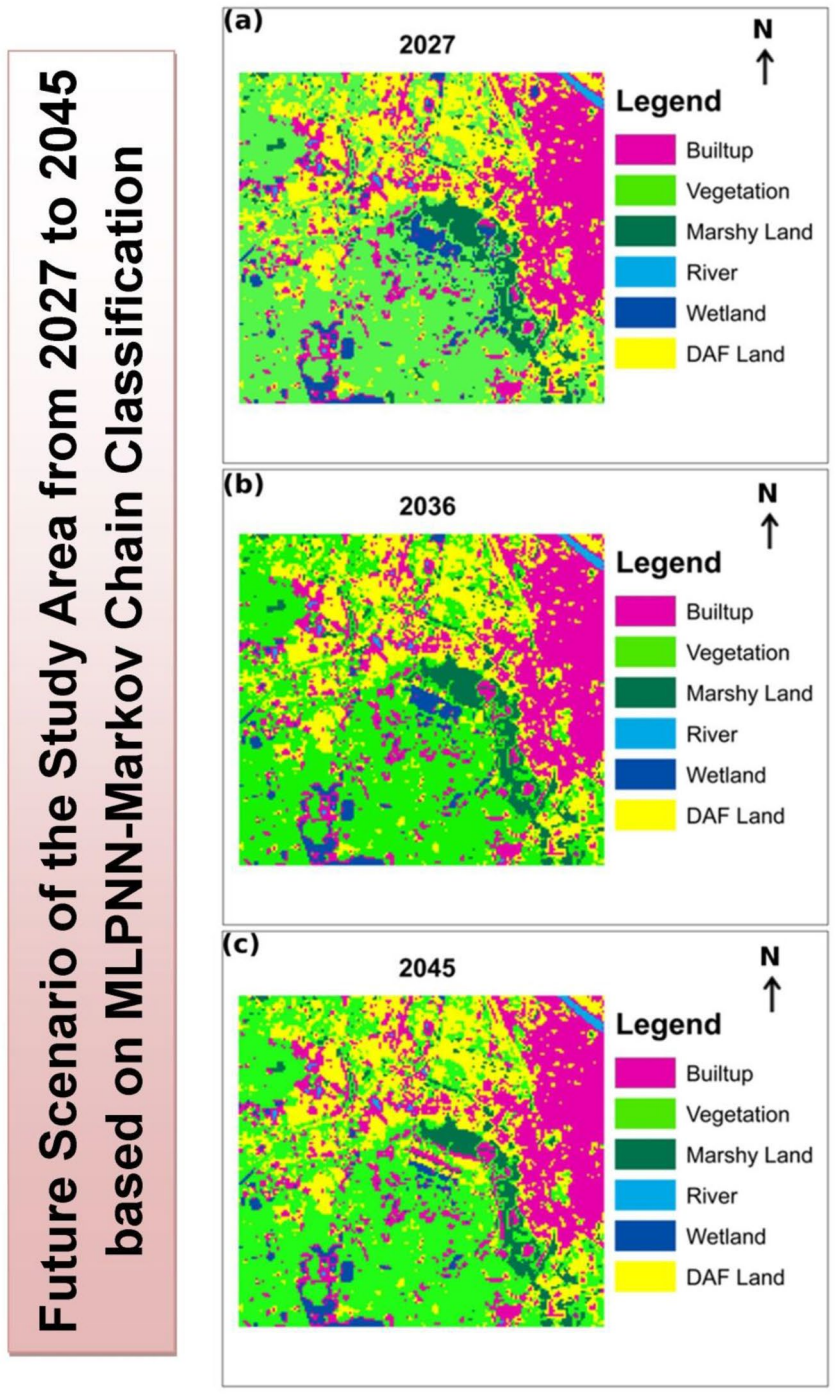
Predicted Land Use and Land Cover (LULC) of Chatra Wetland and its surrounding based on “as usual growth” (**a**) 2027; (**b**) 2045; (**c**) 2045.

After identification of the on-going shrinking and fragmenting character of Chatra Wetland, it is very much essential to project the future scenario of this wetland. Validation procedures like confusion matrix and ROC have displayed more than 80% accuracy (Fig. 5) in predicting future LULC, which suggests good functioning of the model^100^. Besides this, the RMSE was also very low in both cases (RMSE = 12.7% for the first combination and RMSE = 11.8% for the second combination). However, a slightly better validation result has favoured the 2000–2009 LULC combination model to carry out the study (Confusion matrix score 0.82 for the first combination and 0.84 for the second combination). Finally, soft predictions of LULC maps have been simulated for the years 2027, 2036, and 2045 using 2000 as the base year and 2009 as the final year. The predicted LULC for the years 2027, 2036, and 2045 are represented by Fig. 6.

The predicted map of 2027 displays that the area of Chatra Wetland will further decrease and some parts of it will be transformed into built-up and DAF land (Table 11). The area of Chatra Wetland will register a decrease of 6.87% in comparison to 2018 and only 244.62 hectares area will remain as a wetland area. The next phase of the predicted LULC map i.e. 2036 also suggests a similar trend and predicts more areas of Chatra Wetland will be converted into residential and commercial purposes due to the uncontrolled growth of the built-up area. This time the area under Chatra Wetland will remain only 208.26 hectares and the wetland will almost lose its existence by 2045.

**Table 11:** Change in area of different Land Use and Land Cover (LULC) category over the period (2027–2045).

LULC	2027			2036			2045		
	Count	Area (km^2^)	Area in Hectare	Count	Area (km^2^)	Area in Hectare	Count	Area (km^2^)	Area in Hectare
**Future LULC Scenarios based on the proposed Business As Usual (BAU) plan**									
Built-up	6515	5.8635	586.35	6715	6.0435	604.35	6915	6.2235	622.35
Vegetation	11,451	10.3059	1030.59	11,451	10.0029	1000.29	11,451	9.8019	980.19
Marshy land	1759	1.5831	158.31	1536	1.3824	138.24	1313	1.1817	118.17
River	150	0.135	13.5	150	0.135	13.5	150	0.135	13.5
Wetland	959	0.8631	86.31	778	0.7002	70.02	297	0.2673	26.73
DAF	5998	5.3982	539.82	6202	5.8838	588.38	6406	6.2694	626.94
**Future LULC Scenarios based on the proposed environmental plan**									
Built-up	6818	6.1362	613.62	7321	6.5931	659.31	7824	7.0439	704.39
Vegetation	11,451	10.3059	1030.59	11,451	10.3024	1030.24	11,451	10.3054	1030.54
Marshy land	1982	1.7838	178.38	1982	1.7829	178.29	1982	1.7826	178.26
River	150	0.135	13.5	150	0.131	13.1	150	0.134	13.4
Wetland	1140	1.026	102.6	1140	1.0251	102.51	1140	1.0242	102.42
DAF	5291	4.7619	476.19	4788	4.3143	431.43	4285	3.8587	385.87

#### Assessment of loss of ecosystem services from 1991 to 2045

Assessments of ecosystem service values (ESVs) have also confirmed the deterioration of environmental functions in the surrounding region of English Bazar city mainly in the southern direction. On average, the wetland with visual surface water area and swamp area together in 1991 had provided ecosystem services that were worth 61.18 million United States Dollar (USD) /hectare/year. This value increased up to 64.14 million USD/hectare/years in 2000 due to an increase in marshy land. Since then, the environmental services of this region have started to deteriorate due to the conversion of this wetland into other LULC.

As a result, the benefit from different environmental functions such as water supply, food production, water regulation, nutrient cycling, etc. has also been disrupted significantly. This has resulted in comparatively low ESVs in 2009. The total value of ecosystem services, obtained from Chatra Wetland in 2009, was about 18.96 million USD/hectare/year. It shows a 70.44% decrease from the year 2000. However, the area of Chatra Wetland has reduced down further during 2009–2018. During this time, the rate of conversion of Chatra Wetland was due to residential and commercial purposes. As a result, the net value of ecosystem services has also decreased further. The amount of ESVs has registered a reduction of about 7.47% and it came down to 17.55 million USD/hectare/year in 2018.

Ecosystem service values of different environment units for different environmental functions have been given in detail in Appendix 1–10 (see supplementary section). As per the future scenario, the ecosystem services values of this wetland will come down further and it will be only 17.65 million USD/hectare/year in 2027 (Table 12). With further shrinkage of the area of Chatra Wetland, the net value of ESVs from this wetland will remain only 13.36 million USD/hectare/year in 2036 (Table 12) and 6.78 million USD/hectare/year in 2045 (Table 12). It shows a decrease of 88.92% as compared to the ESVs of 1991. The entire area of this wetland will be converted into other LULC as per the prediction of the IDRISI TerrSet software^1^. This will result in the loss of one major environmental unit in this region and ultimately will cause serious environmental deterioration in this region.

**Table 12:** Ecosystem service values for different land use and land cover (LULC) year.

LULC year	Ecosystem service value (US Million$/ha/year)						Total
	Built-up	Vegetation	Marshy land	River	Wetland	Agricultural land	
**Previous year**							
1991	3.05	5.51	6.76	0.19	55.67	1.29	72.47
2000	3.64	2.5	19.86	0.18	44.28	1.54	72
2009	3.79	5.55	4.58	0.17	14.38	2.75	31.22
2018	3.8	4.36	4.13	0.16	13.41	4.11	29.97
**Predicted LULC based on as usual growth**							
2027	3.9	5.55	4.07	0.17	12.1	3.01	28.8
2036	4.03	5.38	3.55	0.16	9.81	3.27	26.2
2045	4.14	5.28	3.04	0.17	3.74	3.49	19.86
**LULC based on proposed environmental plan**							
2027	4.09	5.55	4.58	0.17	14.38	2.65	31.42
2036	4.39	5.54	4.58	0.16	14.37	2.40	31.44
2045	4.69	5.55	4.58	0.17	14.36	2.15	31.5

## Discussion

LULC map of the study area defines six different types of LULC for the years of 2000, 2009, and 2018. The post-monsoon month (November) was taken for the LULC as it will enable the net area loss after the monsoon season^17^. Lean period (dry season) was avoided in this study as some portion of Chatra Wetland gets covered by plantation at this time and it makes it very difficult to assess the actual extent. Analysis of spatial–temporal LULC change displays that expansion of built-up and DAF land within the periphery of Chatra Wetland was the main reason behind its net area loss. It is also a major reason for wetland loss throughout India^101^. The open space of Chatra Wetland in the southern and western sides during the lean period becomes the ground of cultivation. It was observed that the recurrence of cultivation without any restriction has converted the wetland into a permanent plot of agriculture and has led to the loss of wetland in West Bengal^17, 102^. A similar trend was also observed in the case of Chatra Wetland (Pearson’s correlation = 0.92; R^2^ = 0.66 with *p* < 0.001 between net area loss of wetland and expansion of agriculture).

The extension of Chatra Wetland was continuous in 2000 and the entire adjoining part of southwestern English Bazar city was under the swamp and wetland with visual surface water cover. However, with the expansion of English Bazar city, the area under Chatra Wetland has started to squeeze during the successive period^103^.

Conversion of wetland into built-up areas is very much profitable for the housing sector as the land is free and the process is cheap^104–106^. Chatra Wetland as the peri-urban wetland of English Bazar city has also paid the price of urbanization, which has affected the eastern part of this wetland. Further analysis by the landscape metrics also indicates the gradual fragmentation of this wetland over the period.

Several past studies have shown that large patches are more suitable for bigger and greater biodiversity^107, 108^) and some studies have shown the importance of connectivity among the habitat patches within the urban landscape^108^. However, the result of landscape metrics has shown a simultaneous decrease both in the patch size and connectivity. It indicates that larger patches are disaggregating into smaller patches with greater dispersion^58^. This will ultimately harm ecosystem services^26, 84, 109^ and biodiversity as it will influence the species dispersal due to a decrease in habitat connectivity and patch size.

The twenty-first century has registered a huge number of rural to urban migration in India in search of a better living standard^110^. The same situation can also be observed in the case of English Bazar city. However, most of the newly migrated rural people were unable to purchase land within the city due to the high cost of the land value and it forced them to settle in the vicinity of the city. In terms of accessibility, the adjoining areas of the western and southwestern parts of this city are much more suitable to settle down in due to the proximity of National Highway 34 and Malda Town railway station. It acts as the driving force to settle down in this region^39, 75, 103^. This has ultimately resulted in the conversion of wetland into residential and commercial use.

The transition probability scenarios based on 2000 and 2009 LULC illustrates relatively better suitability in prediction. The model suggests that the conversion process of the natural cover of Chatra Wetland is of a centripetal type wherein the peripheral region is first converted into DAF land and later on built-up has emerged in this place. This process gradually proceeds towards the inner core of Chatra Wetland and the model suggests that the existing LULC conversion rate will consume almost the entire Chatra Wetland by 2045.

As a consequence of this, it will bring several negative environmental effects and some of them are already visible^103^. Besides this, Chatra Wetland acts as a central drainage system in this region. If the wetland area has been converted into other LULC, then waterlogging conditions will possibly arise in the western and southwestern part of English Bazar city after heavy rainfall. This situation has already started to take place in this region. An interview of 100 residents by the authors in this region has revealed that the frequency and duration of waterlogging conditions after heavy rainfall has increased around 19% over the last five years as per their opinion. However, still, there is no appropriate action plan in this regard to tackling this emerging problem.

Finally, It is a known fact that wetland provides many environmental functions to urban systems such as flood control, filtration of polluted elements, nutrient recycling, accretion of sediment, recharge of groundwater, erosion rate control, preservation of biotic elements, etc. So, the loss of wetland will result in the loss of ecosystem services^31, 111, 112^. A decrease in the net area of Chatra Wetland in the study area has also resulted in a decrease of 71.90% ecosystem services value from 1991 to 2018. As per the projected LULC, a further 61.35% decrease in ESVs will occur by 2045 if the current LULC dynamics continue. So, the quantitative analysis of Chatra Wetland degradation indicates significant deterioration of this wetland and raises concern for an alternate LULC plan to save this wetland.

## Proposed LULC scenarios following environmental perspective for sustainable development

Peri-urban wetlands are considered an essential component of the urban ecosystem and sustainable use of these units helps to meet the objectives of sustainable development goals^113^. These wetlands help to improve the environmental condition of an urban area by supplying several environmental benefits such as offsetting carbon emission^114^, air pollutants reduction^115^, regulation of microclimate^116^, amenities and recreation^117^. These ultimately help to maintain sustainable urban development^118–121^.

Despite all of these benefits, the degradation of peri-urban/urban wetlands across the world has remained a matter of serious concern. In India, the process of urbanization took place at a much faster rate after the implementation of economic reforms in 1991. As a result, all the existing urban centers have started to expand their area through the development of urban sprawl. It has generated immense pressure on the urban/peri-urban wetlands, which has ultimately resulted in the rapid infilling of these wetlands due to the growing demand for lands^41, 122, 123^.

Ongoing degradation of Chatra Wetland is also a result of this rapid urbanization. It is very much true that the survival of this important wetland is essential not only for the residents but also for the sustainability of this city. In this regard, the authors have tried to present environmental perspective-based LULC projections in the near future without affecting the environmental units. Besides this, the focus was also given to the accessibility of the newly proposed built-up areas while projecting future LULC. The environment based LULC scenarios was prepared using the Land Change Modeler extension of IDRISI TerrSet^1^. In this case, Normalized Difference Water Index (NDWI) ((G − NIR)/(G + NIR)), Normalized Difference Vegetation Index (NDVI) ((NIR − RED)/(NIR + RED)) map was prepared in ArcGIS 10.2.1 for the study area additionally along with the previous layers. Fuzzy membership function ‘small’ was applied in the case of NDWI and NDVI, which represents the absence of these units are suitable for built-up expansion. English Bazar city is an unplanned city. Hence, NDWI and NDVI were not taken into consideration in the previous section of future LULC prediction.

**Figure 7:**
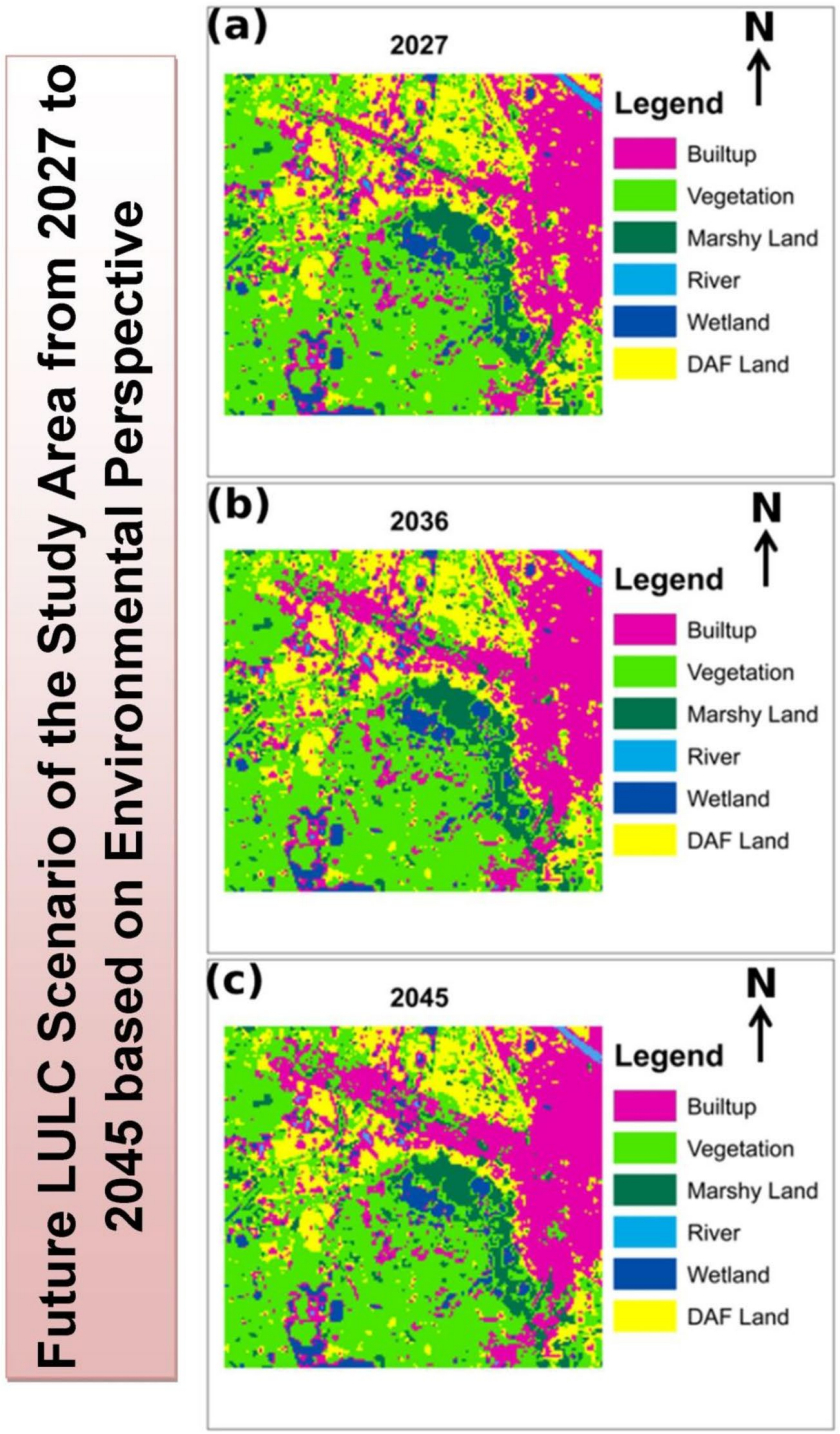
Predicted Land Use and Land Cover (LULC) of Chatra Wetland and its surrounding based on the proposed plan (**a**) 2027; (**b**) 2045; (**c**) 2045^1^.

However, this section deals with the prediction of LULC by protecting the natural environment. So, NDWI and NDVI were taken into consideration. All other criteria remained the same for this modeling. Finally, three LULC maps for the years 2027, 2036, and 2045 respectively (Fig. 7) have been projected. All three LULC maps display suitable areas for built-up expansion in the future without deteriorating the environmental units. Table 11 represents the projected transformation of LULC in the future by this model.

The proposed environment based LULC plan resembles no significant further degradation in the area of Chatra Wetland. Besides this, it also highlights that the area along the state highway-10 towards the North-Western side of the city is much more suitable for future urban expansion both in terms of connectivity and from an environmental perspective. The expansion of urban areas will take place in this region at the expanse of DAF land (mainly fallow land). Table 12 represents the consequently estimated ecosystem service values for these proposed LULCs. It suggests no further significant decrease in the net ecosystem service values of the region. Hence, the proposed LULC model is quite acceptable to implement at the ground level.

## Conclusion

The present study deals with the effects of LULC transformation on environmental units, especially on Chatra Wetland. The quantitative assessment displays over 60% degradation of Chatra Wetland in the last 28 years due to anthropogenic influence. As a result of this, total ESVs from Chatra Wetland has decreased from 61.18 million USD/hectare/year in 1991 to 17.55 million USD/hectare/year in 2018.

The prediction model based on the existing LULC transformation rate shows that there is a probability of complete loss of this wetland within the first half of the twenty-first century. Further assessment of ecosystem service values also suggests significant environmental deterioration in this region. In recent years, the Government of India has promoted the development of smart cities which are eco-friendly. But, the situation in the case of English Bazar city shows opposite condition where the peri-urban Chatra Wetland registered significant deterioration in the past 18 years. Besides this, still, there is no proposed LULC plan in the case of this city for the future. It indicates the possibility that the conversion of Chatra Wetland will continue in the future as well.

In this regard, the authors have tried to present possible areas for future built-up expansion in this region by protecting the environmental units. The proposed LULC map by the authors shows that the expansion of built-up in the northwest side of the city along the major highway will be suitable in the near future. However, a proper action plan from the local administrative unit is required to stop further encroachment in the area of Chatra Wetland.

## Limitations of this study and future scope

Social and economic drivers such as political influence, economic growths, etc. are very important behind the LULC change of any region. However, this study has not incorporated these into LULC modeling due to the non-availability of such databases at the local level and its non-spatial characteristics. Besides this, the study has taken into consideration the MLPNN-Markov chain model for LULC modeling as it comes automatically with the Land Change Modeler. The suitability of other models such as the CA-Markov chain model etc. has not been explored. Finally, the ESVs of Chatra Wetland has been calculated based on global coefficients. However, the estimation of coefficients for local areas may provide slightly better results.

## Supplementary Information

Supplementary Informations

## References

[1] Clarke Labs. IDRISI TerrSet Geospatial Monitoring and Modeling Software. Windows. V18.31 (2020). Website: https://clarklabs.org/terrset/. Download: https://webforpc.net/clark-labs-terrset-idrisi-18-31-free-download.

[2] Wu CY, Chen W (2020). Indicator systemconstruction and health assessment of wetland ecosystem——taking Hongze Lake Wetland China as an example. Ecol. Indic..

[3] Wu CY, Chen W, Cao C, Tian R, Liu D, Bao D (2018). Diagnosis of wetland ecosystem health in the Zoige wetland, Sichuan of China. Wetlands.

[4] The Ramsar Convention Secretariat. The Convention on Wetlands text, as originally adopted in 1971. Annex I of the Final Act of the Ramsar conference in 1971, published in the Proceedings, and registered with UNESCO, the Depositary (1971). https://www.ramsar.org/sites/default/files/documents/library/original_1971_convention_e.pdf.

[5] CBD press brief. Wetlands and ecosystem services. www.cbd.int (2015).

[6] Gopal, B. *Guidelines for Rapid Assessment of Biodiversity and Ecosystem Services of Wetlands*. National Institute of Ecology, version 1.0, 1–24. APN Reference: Project CBA2014‐05NSY(B&ES)‐Gopal (2015).

[7] Muhammed SN, Sabiu N, Khalil MS (2015). An overview of urbanization and its challenges on sustainable development in Nigeria. Dutse J. Pure Appl. Sci..

[8] Padmanaban R, Bhowmik AK (2017). Modelling urban sprawl using remotely sensed data: a case study of Chennai City, Tamilnadu. Entropy.

[9] Tian, G., Xinliang, X. *et al.* The Comparison and Modeling of the Driving Factors of Urban Expansion for Thirty-Five Big Cities in the Three Regions in China. Adv. Meteorol. Volume 2016, Article ID 3109396 (2015).

[10] Hoa T, Dao H, Saborowski J, Hölscher D (2016). Patterns of tree community differences in the core and buffer zones of a nature reserve in north-western Vietnam. Glob. Ecol. Conserv..

[11] Kometa SS (2018). Urban development and its implications on wetland ecosystem services in NDop, Cameroon. Environ. Manag. Sustain. Dev..

[12] Lee SY, Dunn RJK, Young RA (2006). Impact of urbanization on coastal water structure and function. Austral Ecol..

[13] Mao D, Wang Z, Wu J (2018). China’s wetlands loss to urban expansion. Land Degrad. Dev..

[14] Zheng XK, Chunhui L, Huang GH, Yang ZF (2008). Research progress in effects of urbanization on wetland ecosystem in watershed. Wetl. Sci..

[15] Ancog R, Ruzol C (2015). Urbanisation adjacent to a wetland of international importance: the case of Olango Island wildlife sanctuary, metro cebu, Philippines. Habitat Int..

[16] Ghosh, S., Das, A. Urban expansion induced vulnerability assessment of East Kolkata wetland using fuzzy MCDM method. in *Remote Sensing Applications: Society and Environment*, 1–27. 10.1016/j.rsase.2018.10.014 (2019).

[17] Mondal B, Dolui G, Pramanik M, Maity S (2017). Urban expansion and wetland shrinkage estimation using a GIS-based model in East Kolkata Wetland, India. Ecol. Ind..

[18] Amin AKMK, Aminul HM, Mohammad A (2013). Analysis of the wetland degradation around the vicinity of Dhaka city in Bangladesh. Asian J. Water Environ. Pollut..

[19] Hettiarachchi M, Alwis A, Wijekoon S, Athukorale K (2014). Urban wetlands and disaster resilience of Colombo, Sri Lanka. Int. J. Disaster Resil. Built Environ..

[20] Cham DD, Son NT, Minh NQ, Thanh NT, Dung TT (2020). An analysis of shoreline changes using combined multitemporal remote sensing and digital evaluation model. Civ. Eng. J..

[21] Tian R, Cao C, Peng L, Ma G, Bao D, Guo J, Yomwan P (2016). The use of HJ-1A/B satellite data to detect changes in the size of wetlands in response in to a sudden turn from drought to flood in the middle and lower reaches of the Yangtze River system in China. Geomat. Nat. Hazards Risk.

[22] Islam K, Rahman MdF, Jashimuddin M (2018). Modeling land use change using cellular automata and artificial neural network: the case of Chunati Wildlife Sanctuary, Bangladesh. Ecol. Ind..

[23] Tomar, S., Saha, A., Kumari, M., & Somvanshi, S. Land Use and Land Cover change monitoring of sujapur wetland, Uttar Pradesh: using GIS and Remote sensing techniques. in *17th Esri India User Conference 2017* (2017).

[24] Das A, Basu T (2020). Assessment of peri-urban wetland ecological degradation through importance-performance analysis (IPA): a study on Chatra Wetland, India. Ecol. Ind..

[25] Hoque MZ, Cui S, Islam I, Xu L, Tang J (2020). Future impact of land use/land cover changes on ecosystem services in the lower Meghna River estuary, Bangladesh. Sustainability.

[26] Yirsaw E, Wu W, Shi X, Temesgen H, Bekele B (2017). Land Use/land cover change modeling and the prediction of subsequent changes in ecosystem service values in a coastal area of China, the Su-Xi-Chang region. Sustainability.

[27] Hu Z, Wang S, Bai X, Luo G, Li Q, Wu L, Yang Y, Tian S, Li C, Deng Y (2020). Changes in ecosystem service values in karst areas of China. Agric. Ecosyst. Environ..

[28] Talukdar S, Singha P, Shahfahad M, Praveen B, Rahman A (2020). Dynamics of ecosystem services (ESs) in response to land use land cover (LU/LC) changes in the lower Gangetic plain of India. Ecol. Ind..

[29] Das M, Das A (2019). Estimation of Ecosystem Services (EESs) loss due to transformation of Local Climatic Zones (LCZs) in Sriniketan-Santiniketan Planning Area (SSPA)West Bengal, India. Sustain. Cities Soc..

[30] Song W, Deng X (2015). Effects of urbanization-induced cultivated land loss on ecosystem services in the North China Plain. Energies.

[31] Zorrilla-Miras P, Palomo I, Gómez-Bagghetun E (2014). Effects of land-use change on wetland ecosystem services: a case study in the Doñana marshes (SW Spain). Landsc. Urban Plann..

[32] Hegazy IR, Kaloop MR (2015). Monitoring urban growth and land use change detection with GIS and remote sensing techniques in Daqahlia Governorate Egypt. Int. J. Sustain. Built Environ..

[33] Li D, Bou-Zeid E, Baeck ML, Jessup S, Smith JA (2013). Modeling land surface processes and heavy rainfallin urban environments: sensitivity to urban surface representations. J. Hydrometeor..

[34] Lopez, R.D., Heggem, D.T., Sutton, D., *et al.*. Using landscape metrics to develop indicators of great lakes coastal wetland condition. United States Environmental Protection Agency (EPA/600/X-06/002), Office of Research and Development, Office of Water, Washington (2006).

[35] Ma Y, Xu R (2010). Remote sensing monitoring and driving force analysis of urban expansion in Guangzhou City, China. Habitat Int..

[36] Megahed Y, Cabral P, Silva J, Caetano M (2015). Land cover mapping analysis and urban growth modelling using remote sensing techniques in Greater Cairo Region—Egypt. Int. J. Geo-inf..

[37] Costanza R, de Groot R, Sutton P, Van der Ploeg S, Anderson SJ, Kubiszewski I (2014). Changes in the global value of ecosystem services. Glob. Environ. Change.

[38] Sarkar, R. Urbanization in Malda in the Colonial Period Growth of English Bazar as a Case Study 1813 to 1947. Department of History, University of North Bengal. http://hdl.handle.net/10603/165865 (2015).

[39] Shaw R, Das A (2018). Identifying peri-urban growth in small and medium towns using GIS and remote sensing technique: a case study of English Bazar Urban Agglomeration, West Bengal, India. Egypt. J. Remote Sens. Space Sci..

[40] Census of India https://censusindia.gov.in/2011-common/censusdata2011.html (2011).

[41] Ziaul S, Pal S (2017). Estimating wetland insecurity index for Chatra Wetland adjacent English Bazar Municipality of West Bengal. Spat. Inf. Res..

[42] Malda District Magistrate. Malda District action plan handbook. http://wbdmd.gov.in/writereaddata/uploaded/DP/Malda.pdf (2018). Accessed 26 Aug 2020.

[43] Dutta S, Sengupta A (2015). Wetland Restoration, a need for sustenance: a case study Chatra Beel of English Bazar, District Malda, W.B., India. Int. J. Appl. Res..

[44] Kar SK (2018). State of wetland transformation and ecological concerns—a case study of Chatra Wetland, English Bazar, West Bengal. Int. J. Res. Geogr..

[45] Kar SK, Pal S (2012). Changing land use pattern in Chatra wetland of English Bazar Town: rationale and flaws. Int. J. Humanit. Soc. Sci..

[46] ESRI. ArcGIS Desktop: Release 10.2.1. Redlands, CA: Environmental Systems Research Institute. Download: https://support.esri.com/en/download/7462 (2014).

[47] Hurskainen P, Adhikari H, Siljander M, Pellikka PKE, Hemp A (2019). Auxiliary datasets improve accuracy of object-based land use/land cover classification in heterogeneous savanna landscapes. Remote Sens. Environ..

[48] Naikoo MW, Rihan M, Ishtiaque M (2020). Analyses of land use land cover (LULC) change and built-up expansion in the suburb of a metropolitan city: spatio-temporal analysis of Delhi NCR using landsat datasets. J. Urban Manag..

[49] Mohajane M, Essahlaoui A, Oodija F (2018). Land use/land cover (LULC) using landsat data series (MSS, TM, ETM+ and OLI) in Azrou forest, in the central middle Atlas of Morocco. Environments.

[50] Noi PT, Kappas M (2018). Comparison of random forest, k-nearest neighbor, and support vector machine classifiers for land cover classification using sentinel-2 imagery. Sensors.

[51] Raczko E, Zagajewski B (2017). Comparison of support vector machine, random forest and neural network classifiers for tree species classification on airborne hyperspectral APEX images. Eur. Jo. Remote Sens..

[52] Ahmed B, Ahmed R, Zhu X (2013). Evaluation of model validation in land cover dynamics. ISPRS Int. J. Geo-Inf..

[53] Saharan, M. A., Vyas, N., Borana, S. L., & Yadav, S. K. Classification and assessment of the land use–land cover changes in Jodhpur City using remote sensing technologies. *Int. Arch. Photogramm. Remote Sens. Spatial Inf. Sci.*, **1**(5), 767–771. 10.5194/isprs-archives-XLII-5-767-2018 (2019).

[54] R Core Team. R: A language and environment for statistical computing. R Foundation for Statistical Computing, Vienna, Austria. Website: http://www.R-project.org/; R statistical software v4.0.3, Download: https://cran.r-project.org/bin/windows/base/rtest.html (2020).

[55] Huang L, Song J, Yu X, Fang L (2019). Unmanned aerial vehicle remote sensing image segmentation method by combining superpixels with multi-features distance measure. IOP Conf. Seri. Earth Environ. Sci..

[56] Morales-Barquero L, Lyons MB, Phinn SR, Roelfsema CM (2019). Trends in remote sensing accuracy assessment approaches in the context of natural resources. Remote Sens..

[57] Cochran WG (1977). Sampling Techniques.

[58] Haas J, Furberg D, Ban Y (2015). Satellite monitoring of urbanization and environmental impacts—a comparison of Stockholm and Shanghai. Int. J. Appl. Earth Obs. Geoinf..

[59] Hu, Y., Ban, Y., Zhang, Q., & Liu, J. The trajectory of urbanization process in the Yangtze River Delta during 1990 to 2005. in *Joint Urban Remote Sensing Event*, 1–8 (2009).

[60] He X, Gao Y, Niu J, Zhao Y (2011). Landscape pattern changes under the impacts of urbanization in the yellow river wetland-taking Zhengzhoub as an example. Proc. Environ. Sci..

[61] Meng L, Dong J (2019). LULC and ecosystem services value assessment for wetlands: a case study in Nansi Lake, China. Water.

[62] Tamagnone P, Comino E, Rosso M (2020). Landscape metrics integrated in hydraulic modeling for river restoration planning. Environ. Model. Assess..

[63] Uuemaa, E., Antrop, M., Roosaare, J., Marja, R., *et al.* Landscape metrics and indices: an overview of their use in landscape research. *Living Rev. Landsc. Res.* **3**, 1 (2009).

[64] McGarigal, K. & Marks, B. FRAGSTATS: spatial pattern analysis program for quantifying landscape structure. General Technical Reports (U.S. Department of Agriculture, Forest Service, Pacific Northwest Research Station, Portland, 1995).

[65] Cook NR (2007). Use and misuse of the receiver operating characteristic curve in risk prediction. Circulation.

[66] Serra P, Pons X, Sauri D (2008). Land-cover and land-use change in a Mediterranean landscape: a spatial analysis of driving forces integrating biophysical and human factors. Appl. Geogr..

[67] Olofsson P, Foody GM, Herold M (2014). Good practices for estimating area and assessing accuracy of land change. Remote Sens. Environ..

[68] Intellipaat. Confusion Matrix | How to Implement Confusion Matrix In R | Intellipaat. YouTube. https://www.youtube.com/watch?v=CuJc1MFY23k (2019).

[69] Basu T, Pal S (2019). A GIS-based factor clustering and landslide susceptibility analysis using AHP for Gish River Basin, India. Environ. Dev. Sustain..

[70] Hu X, Weng Q (2009). Estimating impervious surfaces from medium spatial resolution imagery using the self-organizing map and multi-layer perceptron neural networks. Remote Sens. Environ..

[71] Thapa RB, Murayama Y (2012). Scenario based urban growth allocation in Kathmandu Valley, Nepal. Landsc. Urban Plann..

[72] Deng Z, Zhang X, Pan G (2015). Simulation of land use/land cover change and its effects on the hydrological characteristics of the upper reaches of the Hanjiang Basin. Environ. Earth Sci..

[73] Liu G, Chen S, Gu J (2019). Urban renewal simulation with spatial, economic and policy dynamics: the rent-gap theory-based model and the case study of Chongqing. Land Use Policy.

[74] Yue W, Liu Y, Fan P (2013). Measuring urban sprawl and its drivers in large Chinese cities: the case of Hangzhou. Land Use Policy.

[75] Dutta I, Das A (2019). Modeling dynamics of peri-urban interface based on principal component analysis (PCA) and cluster analysis (CA): a study of English Bazar Urban Agglomeration, West Bengal. Model. Earth Syst. Environ..

[76] Basu T, Pal S (2018). RS-GIS based morphometrical and geological multi-criteria approach to the landslide susceptibility mapping in Gish River Basin, West Bengal, India. Adv. Space Res..

[77] Semlali I, Ouadif L, Bahi L (2019). Landslide susceptibility mapping using the analytical hierarchy process and GIS. Curr. Sci..

[78] Sarkar S, Parihar SM, Dutta A (2016). Environmental modelling & software fuzzy risk assessment modelling of east Kolkata wetland area: a remote sensing and GIS based approach. Environ. Modell. Softw..

[79] Shafizadeh-Moghadam H, Helbich M (2013). Spatiotemporal urbanization processes in the megacity of Mumbai, India: a Markov chains-cellular automata urban growth model. Appl. Geogr..

[80] McBratney, A. B., Odeh, I. O. A. Application of fuzzy sets in soil science: fuzzy logic, fuzzy measurements and fuzzy decisions. Geoderma **77**(2–4), 85–113. 10.1016/S0016-7061(97)00017-7. ISSN 0016–7061 (1997).

[81] Tsoukalas LH, Uhrig RE (1997). Fuzzy and Neural Approaches in Engineering.

[82] Lou X, Dimitrakopoulos R (2003). Data-driven fuzzy analysis in quantitative mineral assessment. Comput. Geosci..

[83] Kamwi JM, Cho MA, Kaetsch C, Manda SO, Graz FP, Chirwa PW (2018). Assessing the spatial drivers of land use and land cover change in the protected and communal areas of the Zambezi Region, Namibia. Land.

[84] Lin X, Xu M, Cao C, Singh RP, Chen W, Ju H (2018). Land-use/land-cover changes and their influence on the ecosystem in Chengdu City, China during the period of 1992–2018. Sustainability.

[85] Reilly MK, O’Mara MP, Seto KC (2009). From Bangalore to the bay area: comparing transportation and activity accessibility as drivers of urban growth. Landsc. Urban Plann..

[86] Kavzoglu T, Mather PM (2003). The use of back-propagation artificial neural networks in land cover classification. Int. J. Remote Sens..

[87] Almeida CM, Gleriani JM, Castejon EF, Soares-Filho BS (2008). Using neural networks and cellular automata for modeling intra-urban land-use dynamics. Int. J. Geogr. Inf. Sci..

[88] Chim K, Tunnicliffe J, Shamseldin A, Ota T (2019). Land use change detection and prediction in upper Siem Reap River, Cambodia. Hydrology.

[89] Yeh AG, Li X (2003). Simulation of development alternatives using neural networks, cellular automata, and GIS for urban planning. Photogramm. Eng. Remote Sens..

[90] Ansari A, Golabi MH (2019). Prediction of spatial land use changes based on LCM in a GIS environment for Desert Wetlands—a case study: Meighan Wetland, Iran. Int. Soil Water Conserv. Res..

[91] Eastman, J. R., & Jiang, H. Fuzzy measures in multi-criteria evaluation. in *Proceedings, Second International Symposium on Spatial Accuracy Assessment in Natural Resources and Environmental Studies* 527–534. (Fort Collins, GIS World Inc., 1996).

[92] Costanza R, Arge R, Groot R (1997). The value of the world’s ecosystem services and natural capital. Nature.

[93] Lyu R, Zhang J, Xu M, Li J (2018). Impacts of urbanization on ecosystem services and their temporal relations: a case study in Northern Ningxia, China. Land Use Policy.

[94] Li J, Chen H, Zhang C, Pan T (2019). Variations in ecosystem service value in response to landuse/land cover changes in Central Asia from 1995–2035. PeerJ.

[95] Yi H, Güneralp B, Filippi AM, Kreuter UP, Güneralp I (2017). Impacts of land change on ecosystem services in the San Antonio River Basin, Texas, from 1984 to 2010. Ecol. Econ..

[96] de Groot RS, Brander L, Ploeg S, Costanza R, Bernard F, Braat L (2012). Global estimates of the value of ecosystems and their services in monetary units. Ecosyst. Serv..

[97] Iqbal S (2010). Flood and erosion induced population displacements: a socio-economic case study in the Gangetic Riverine tract at Malda District, West Bengal, India. J. Hum. Ecol..

[98] Ahmad, T., Pandey, A.C., & Kumar, A. Impact of flooding on land use/land cover transformation in Wular lake and its environs, Kashmir valley, India using geoinformatics. in *ISPRS Annals of the Photogrammetry, Remote Sensing and Spatial Information Sciences*, vol. IV-4/W4. 10.5194/isprs-annals-IV-4-W4-13-2017 (2017).

[99] Nandargi SS, Barman K (2018). Evaluation of climate change impact on rainfall variation in West Bengal. Acta Sci Agric.

[100] Rasyid AR, Bhandary NP, Ryuichi Y (2016). Performance of frequency ratio and logistic regression model in creating GIS based landslides susceptibility map at Lompobattang Mountain, Indonesia. Geoenviron. Disasters.

[101] Foote AL, Pandey S, Krogman NT (1996). Processes of wetland loss in India. Environ. Conserv..

[102] Li, Z., Wang, Z., & Pan, B. Wetland ecosystems of the Yellow River source zone. in *Landscape and Ecosystem Diversity, Dynamics and Management in the Yellow River Source Zone*, 183–207 (Springer, Cham, 2016).

[103] Pal S, Ziaul Sk (2017). Detection of Land Use and Land Cover change and land surface temperature in English Bazar urban centre. Egypt. J. Remote Sens. Space Sci..

[104] Asselen SV, Verburg PH, Vermaat JE, Janse JH (2013). Drivers of wetland conservation: a global meta-analysis. PLoS ONE.

[105] Bassi N, Kumar MD, Sharma A, Pardha-Saradhi P (2014). Status of wetlands in India: a review of extent, ecosystem benefits, threats and management strategies. J. Hydrol. Reg. Stud..

[106] Ramachandra TV, Aithal BH, Sanna DD (2012). Insights to urban dynamics through landscape spatial pattern analysis. Int. J. Appl. Earth Obs. Geoinf..

[107] Evans G (2009). Creative cities, creative spaces and urban policy. Urban Stud..

[108] Magle SB, Theobald DM, Crooks KR (2009). A comparison of metrics predicting landscape connectivity for a highly interactive species along an urban gradient in Colorado, USA. Landsc. Ecol..

[109] Lin S, Wu R, Yang F, Wang J, Wu W (2018). Spatial trade-offs and synergies among ecosystem services within a global biodiversity hotspot. Ecol. Ind..

[110] Kumar J, Radha G (2017). Social consequences of rural to urban migration: a case of district Udhampur. Asian J. Res. Soc. Sci. Humanit..

[111] Ayanlade A, Proske U (2016). Assessing wetland degradation and loss of ecosystem services in the Niger Delta, Nigeria. Mar. Freshw. Res..

[112] Zhao M, He Z (2018). Evaluation of the effects of land cover change on ecosystem service values in the upper reaches of the Heihe River Basin, Northwestern China. Sustainability.

[113] UNDP (2015). https://www.undp.org/content/dam/undp/library/Environment%20and%20Energy/Ecosystems%20and%20Biodiversity/UNDP%20Stories%20in%20Wetlands28May2015_new.pdf. Accessed 26 Aug 2020.

[114] Melton JR, Wania R, Hodson EL (2013). Present state of global wetland extent and wetland methane modelling: conclusions from a model inter-comparison project (WETCHIMP). Biogeosciences.

[115] Nakayama T (2017). Development of an advanced eco-hydrologic and biogeochemical coupling model aimed at clarifying the missing role of inland water in the global biogeochemical cycle. J. Geophys. Res. Solid Earth.

[116] Dixon MJR, Loh J, Davidson NC, Walpole MJ (2016). Tracking global change in ecosystem area: The Wetland Extent Trends Index. Biol. Conserv..

[117] Carpenter SR, Bennett EM (2011). Reconsideration of the planetary boundary for phosphorus. Environ. Res. Lett..

[118] Chen WY, Jim CY (2008). Assessment and valuation of the ecosystem services provided by urban forests. Ecol. Plan. Manag. Urban For.ests.

[119] Jensen MB, Persson B, Guldager S, Reeh U, Nilsson K (2000). Green structure and sustainability—developing a tool for local planning. Landsc. Urban Plann..

[120] Li J, Sun H, Xing DX, Wang XG (2003). Characteristics of wetland and its conservation in arid and semi-arid areas in Northwest of China. J. Desert Res..

[121] Miller RL, Fujii R (2009). Plant community, primary productivity, and environmental conditions following wetland re-establishment in the Sacramento-San Joaquin Delta, California. Wetl. Ecol. Manag..

[122] Patra S, Sahoo S, Mishra P, Mahapatra SC (2018). Impacts of urbanization on land use/cover changes and its probable implications on local climate and groundwater level. J. Urban Manag..

[123] Samal DR, Gedam SS (2015). Monitoring land use changes associated with urbanization: an object based image analysis approach. Eur. J. Remote Sens..

