# Author Correction: Development of an integrated peri-urban wetland degradation assessment approach for the Chatra Wetland in eastern India

**DOI:** 10.1038/s41598-021-86634-z

**Published:** 2021-03-24

**Authors:** Tirthankar Basu, Arijit Das, Quoc Bao Pham, Nadhir Al-Ansari, Nguyen Thi Thuy Linh, Gareth Lagerwall

**Affiliations:** 1 Department of Geography, University of Gour Banga, Malda, West Bengal 732103 India; 2 Environmental Quality, Atmospheric Science and Climate Change Research Group, Ton Duc Thang University, Ho Chi Minh City, Vietnam; 3 Faculty of Environment and Labour Safety, Ton Duc Thang University, Ho Chi Minh City, Vietnam; 4 Department of Civil, Environmental and Natural Resources Engineering, Lulea University of Technology, 97187 Luleå, Sweden; 5 Thuyloi University, 175 Tay Son, Dong Da, Hanoi, Vietnam; 6 Bioresources Engineering, School of Engineering, University of KwaZulu-Natal, Scottsville, P. Bag X01, Pietermaritzburg, 3209 Republic of South Africa; 7 The Centre for Water Resources Research, University of KwaZulu-Natal, Scottsville, P. Bag X01, Pietermaritzburg, 3209 Republic of South Africa

Correction to: *Scientific Reports* 10.1038/s41598-021-83512-6, published online 24 February 2021

In the original version of this Article, Nguyen Thi Thuy Linh was incorrectly affiliated with ‘Institute of Research and Development, Duy Tan University, Danang 550000, Vietnam’ and ‘Faculty of Environmental and Chemical Engineering, Duy Tan University, Danang 550000, Vietnam’. The correct affiliation is listed below.

Thuyloi University, 175 Tay Son, Dong Da, Hanoi, Vietnam.

Additionally, the corresponding email of Nguyen Thi Thuy Linh was incorrect. Correspondence and request for materials should be addressed to linhntt@tlu.edu.vn.

These errors have now been corrected in the HTML and PDF versions of this Article, and in the accompanying Supplementary Information file.

