# Supplementary material for: Development of an integrated peri-urban wetland degradation assessment approach for the Chatra Wetland in eastern India
Source: Sci Rep. 2021 Feb 24;11:4470. doi: 10.1038/s41598-021-83512-6 (PMC7904909; doi:10.1038/s41598-021-83512-6)
Supplement: Supplementary file 1 — Supplementary Informations [file 41598_2021_83512_MOESM1_ESM.docx]

**Development of an Integrated Peri-Urban Wetland Degradation Assessment Approach** **for the Chatra Wetland in Eastern India**

**Tirthankar Basu^1^, Arijit Das^1^, Quoc Bao Pham^2,3^, Nadhir Al-Ansari^4^, Nguyen Thi Thuy Linh^5^*, Gareth Lagerwall^6,7^**

^1^Deptt. of Geography, University of Gour Banga, Malda 732103, West Bengal, India

^2^Environmental Quality, Atmospheric Science and Climate Change Research Group, Ton Duc Thang University, Ho Chi Minh City, Vietnam

^3^Faculty of Environment and Labour Safety, Ton Duc Thang University, Ho Chi Minh City, Vietnam

^4^Department of Civil, Environmental and Natural Resources Engineering, Lulea University of Technology, 97187, Lulea, Sweden

^5^Thuyloi University, 175 Tay Son, Dong Da, Hanoi, Vietnam

^6^Bioresources Engineering, School of Engineering, University of KwaZulu-Natal, P. Bag X01, Scottsville 3209, Pietermaritzburg, Republic of South Africa

^7^The Centre for Water Resources Research, University of KwaZulu-Natal, Scottsville, P. Bag X01, Pietermaritzburg 3209, Republic of South Africa

*Corresponding author: linhntt@tlu.edu.vn


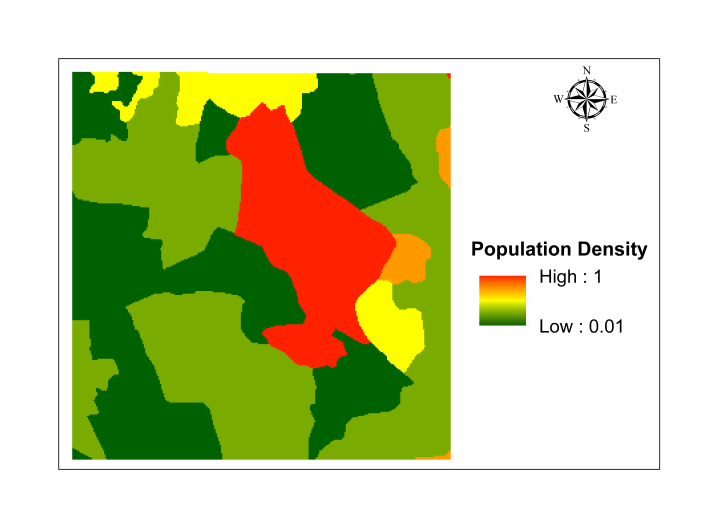

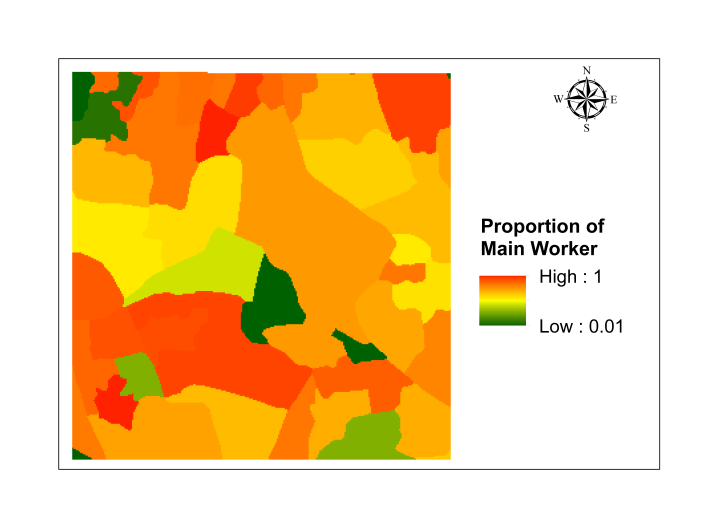

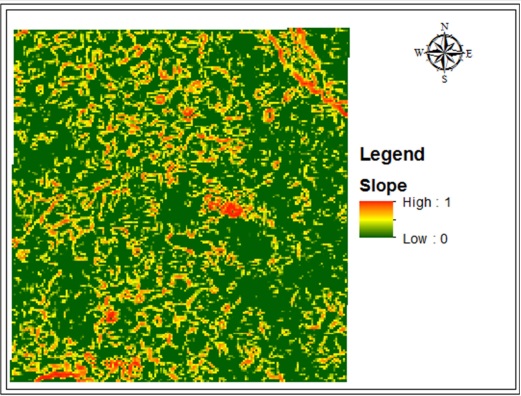

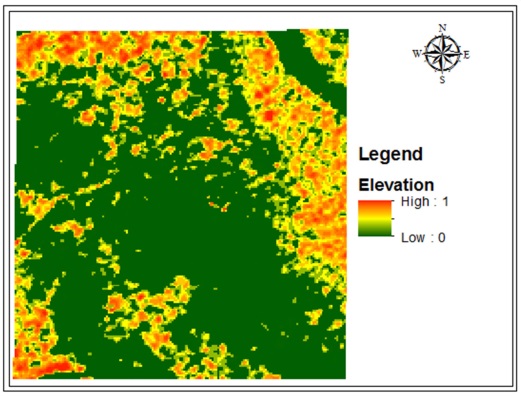


(d)

(c)

(b)

(a)


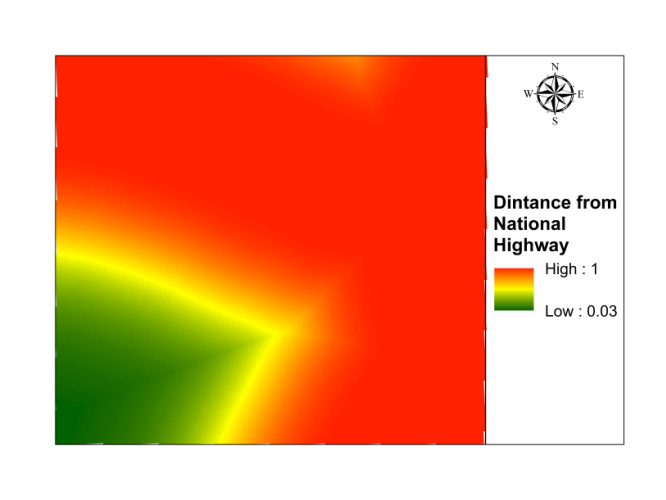

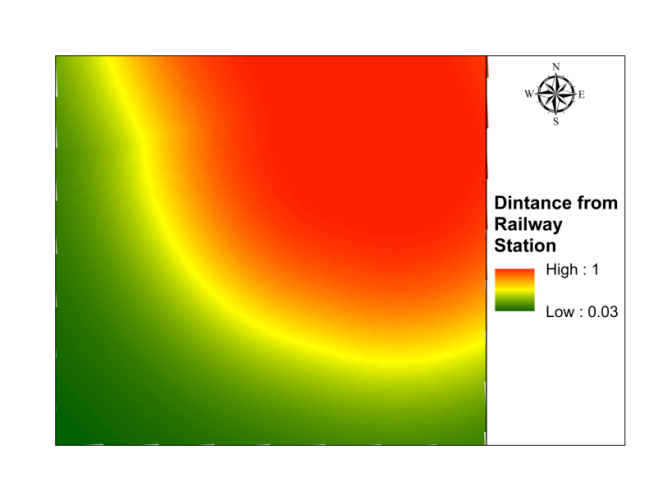


(f)

(e)


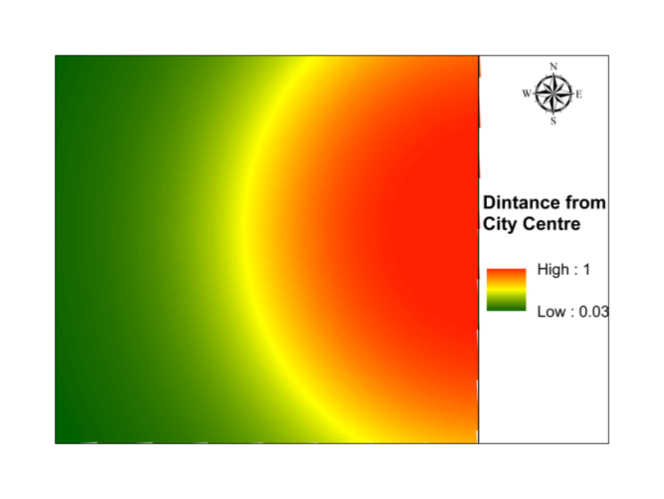

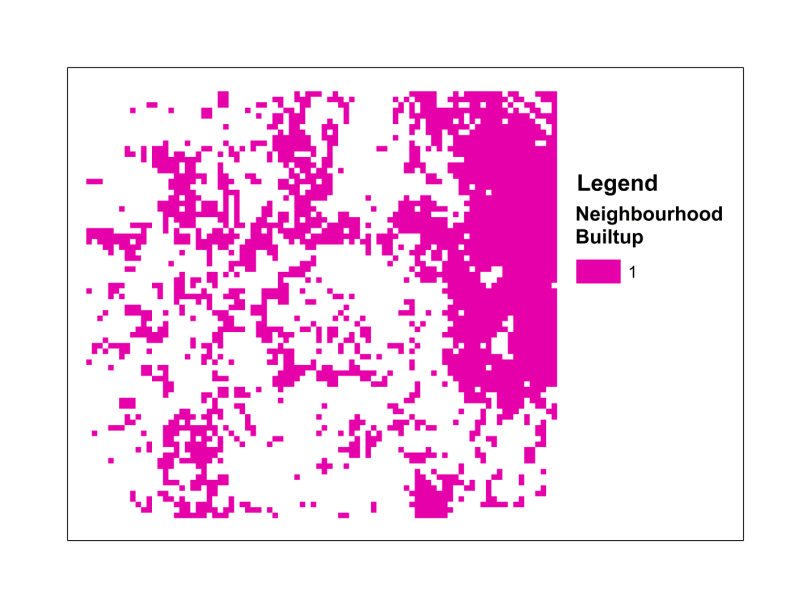


(h)

(g)


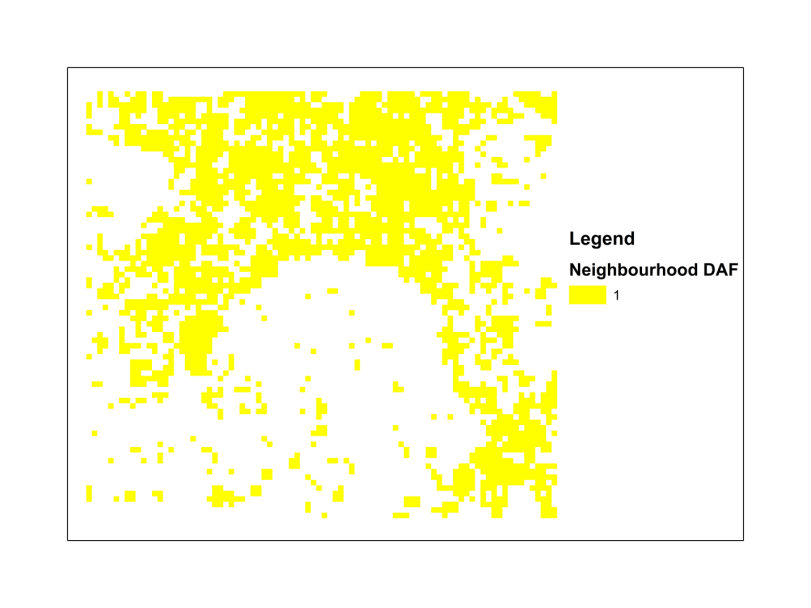


(i)

Figure S1: Fuzzy standardization on different variable (a) Population Density; (b) Proportion of main worker; (c) Slope; (d) Elevation; (e) Distance from National Highway; (f) Distance from railway station; (g) Distance from city centre; (h) Neighbourhood built-up factor; (i) Neighbourhood DAF factor

Appendix 1: Ecosystem service value for different LULC for the year of 1991

| **Ecosystem Services** | **Ecosystem Service value (US Million$/ha./year) for 1991** | | | | | |
| --- | --- | --- | --- | --- | --- | --- |
|  | **Built-up** | **Vegetation** | **Marshy land** | **River** | **Wetland** | **DAF land** |
| **Provisional services** |  |  |  |  |  |  |
| Water supply | 0.00 | 0.03 | 0.11 | 0.03 | 0.38 | 0.09 |
| Food production | 0.00 | 0.20 | 0.16 | 0.001 | 0.38 | 0.54 |
| Raw materials | 0.00 | 0.09 | 0.14 | 0.00 | 0.17 | 0.05 |
| Genetic resources | 0.00 | 1.55 | 0.03 | 0.00 | 0.10 | 0.24 |
| **Regulating services** |  |  |  |  |  |  |
| Water regulation | 0.01 | 0.01 | 1.48 | 0.12 | 0.71 | 0 |
| Waste treatment | 0.00 | 0.12 | 0.79 | 0.01 | 44.22 | 0.09 |
| Erosion control | 0.00 | 0.35 | 0.69 | 0.00 | 1.39 | 0.03 |
| Climate regulation | 0.41 | 2.09 | 0.13 | 0.00 | 0.08 | 0.10 |
| Biological control | 0.00 | 0.01 | 0.25 | 0.00 | 0.12 | 0.01 |
| Gas regulation | 0.00 | 0.01 | 0.00 | 0.00 | 0.00 | 0.00 |
| Disturbance regulation | 0.00 | 0.07 | 0.79 | 0.00 | 1.83 | 0.00 |
| **Supporting services** |  |  |  |  |  |  |
| Nutrient cycling | 0.00 | 0.00 | 0.45 | 0.00 | 0.23 | 0.00 |
| Pollution | 0.00 | 0.03 | 0.00 | 0.00 | 0.00 | 0.01 |
| Soil formation | 0.00 | 0.01 | 0.00 | 0.00 | 0.00 | 0.12 |
| Habitat/Refugio | 0.00 | 0.04 | 0.65 | 0.00 | 4.95 | 0.00 |
| **Cultural services** |  |  |  |  |  |  |
| Recreation | 2.62 | 0.89 | 0.58 | 0.03 | 0.87 | 0.02 |
| Culture | 0.00 | 0.00 | 0.52 | 0.00 | 0.25 | 0.00 |
| **Total** | **3.05** | **5.51** | **6.76** | **0.19** | **55.67** | **1.29** |

Appendix 2: Ecosystem service value for different LULC for the year of 2000

| **Ecosystem Services** | **Ecosystem Service value (US Million$/ha./year) for 2000** | | | | | |
| --- | --- | --- | --- | --- | --- | --- |
|  | **Built-up** | **Vegetation** | **Marshy land** | **River** | **Wetland** | **DAF land** |
| **Provisional services** |  |  |  |  |  |  |
| Water supply | 0.00 | 0.01 | 0.32 | 0.03 | 0.30 | 0.11 |
| 0.03 | 0.00 | 0.09 | 0.47 | 0.00 | 0.30 | 0.64 |
| 0.001 | 0.00 | 0.04 | 0.42 | 0.00 | 0.13 | 0.06 |
| 0.00 | 0.00 | 0.71 | 0.08 | 0.00 | 0.08 | 0.29 |
| 0.00 |  |  |  |  |  |  |
|  | 0.01 | 0.00 | 4.34 | 0.12 | 0.57 | 0 |
| 0.12 | 0.00 | 0.06 | 2.33 | 0.01 | 35.17 | 0.11 |
| 0.01 | 0.00 | 0.16 | 2.02 | 0.00 | 1.11 | 0.03 |
| 0.00 | 0.49 | 0.95 | 0.38 | 0.00 | 0.06 | 0.11 |
| 0.00 | 0.00 | 0.01 | 0.73 | 0.00 | 0.10 | 0.01 |
| 0.00 | 0.00 | 0.01 | 0.00 | 0.00 | 0.00 | 0 |
| 0.00 | 0.00 | 0.03 | 2.31 | 0.00 | 1.45 | 0 |
| 0.00 |  |  |  |  |  |  |
|  | 0.00 | 0.00 | 1.32 | 0.00 | 0.18 | 0 |
| 0.00 | 0.00 | 0.01 | 0.00 | 0.00 | 0.00 | 0.01 |
| 0.00 | 0.00 | 0.01 | 0.00 | 0.00 | 0.00 | 0.15 |
| 0.00 | 0.00 | 0.02 | 1.90 | 0.00 | 3.93 | 0 |
| 0.00 |  |  |  |  |  |  |
|  | 3.14 | 0.40 | 1.71 | 0.03 | 0.69 | 0.02 |
| 0.03 | 0.00 | 0.00 | 1.54 | 0.00 | 0.20 | 0 |
| 0.00 | **3.64** | **2.50** | **19.86** | **0.18** | **44.28** | **1.54** |

Appendix 3: Ecosystem service value for different LULC for the year of 2009

| **Ecosystem Services** | **Ecosystem Service value (US Million$/ha./year) for 2009** | | | | | |
| --- | --- | --- | --- | --- | --- | --- |
|  | **Built-up** | **Vegetation** | **Marshy land** | **River** | **Wetland** | **DAF land** |
| **Provisional services** |  |  |  |  |  |  |
| Water supply | 0.00 | 0.03 | 0.07 | 0.02 | 0.10 | 0.20 |
| Food production | 0.00 | 0.21 | 0.11 | 0.00 | 0.10 | 0.15 |
| Raw materials | 0.00 | 0.09 | 0.10 | 0.00 | 0.04 | 0.11 |
| Genetic resources | 0.00 | 1.56 | 0.02 | 0.00 | 0.02 | 0.52 |
| **Regulating services** |  |  |  |  |  |  |
| Water regulation | 0.01 | 0.01 | 1.00 | 0.10 | 0.18 | 0 |
| Waste treatment | 0.00 | 0.12 | 0.54 | 0.01 | 11.42 | 0.20 |
| Erosion control | 0.00 | 0.35 | 0.47 | 0.00 | 0.36 | 0.05 |
| Climate regulation | 0.51 | 2.11 | 0.09 | 0.00 | 0.02 | 0.20 |
| Biological control | 0.00 | 0.01 | 0.17 | 0.00 | 0.03 | 0.02 |
| Gas regulation | 0.00 | 0.01 | 0.00 | 0.00 | 0.00 | 0 |
| Disturbance regulation | 0.00 | 0.07 | 0.53 | 0.00 | 0.47 | 0 |
| **Supporting services** |  |  |  |  |  |  |
| Nutrient cycling | 0.00 | 0.00 | 0.31 | 0.00 | 0.06 | 0 |
| Pollution | 0.00 | 0.03 | 0.00 | 0.00 | 0.00 | 0.01 |
| Soil formation | 0.00 | 0.01 | 0.00 | 0.00 | 0.00 | 0.26 |
| Habitat/Refugio | 0.00 | 0.04 | 0.44 | 0.00 | 1.28 | 0 |
| **Cultural services** |  |  |  |  |  |  |
| Recreation | 3.26 | 0.89 | 0.39 | 0.03 | 0.23 | 0.41 |
| Culture | 0.00 | 0.00 | 0.36 | 0.00 | 0.07 | 0 |
| **Total** | **3.79** | **5.55** | **4.58** | **0.17** | **14.38** | **2.75** |

Appendix 4: Ecosystem service value for different LULC for the year of 2018

| **Ecosystem Services** | **Ecosystem Service value (US Million$/ha./year) for 2018** | | | | | |
| --- | --- | --- | --- | --- | --- | --- |
|  | **Built-up** | **Vegetation** | **Marshy land** | **River** | **Wetland** | **DAF land** |
| **Provisional services** |  |  |  |  |  |  |
| Water supply | 0.00 | 0.02 | 0.07 | 0.02 | 0.10 | 0.30 |
| Food production | 0.00 | 0.16 | 0.10 | 0.00 | 0.10 | 1.71 |
| Raw materials | 0.00 | 0.07 | 0.09 | 0.00 | 0.04 | 0.16 |
| Genetic resources | 0.00 | 1.23 | 0.02 | 0.00 | 0.02 | 0.77 |
| **Regulating services** |  |  |  |  |  |  |
| Water regulation | 0.01 | 0.01 | 0.90 | 0.10 | 0.18 | 0 |
| Waste treatment | 0.00 | 0.10 | 0.49 | 0.01 | 10.48 | 0.29 |
| Erosion control | 0.00 | 0.27 | 0.42 | 0.00 | 0.36 | 0.08 |
| Climate regulation | 0.52 | 1.66 | 0.08 | 0.00 | 0.02 | 0.30 |
| Biological control | 0.00 | 0.01 | 0.15 | 0.00 | 0.03 | 0.02 |
| Gas regulation | 0.00 | 0.01 | 0.00 | 0.00 | 0.00 | 0 |
| Disturbance regulation | 0.00 | 0.05 | 0.48 | 0.00 | 0.47 | 0 |
| **Supporting services** |  |  |  |  |  |  |
| Nutrient cycling | 0.00 | 0.00 | 0.28 | 0.00 | 0.06 | 0 |
| Pollution | 0.00 | 0.02 | 0.00 | 0.00 | 0.00 | 0.02 |
| Soil formation | 0.00 | 0.01 | 0.00 | 0.00 | 0.00 | 0.39 |
| Habitat/Refugio | 0.00 | 0.03 | 0.40 | 0.00 | 1.27 | 0 |
| **Cultural services** |  |  |  |  |  |  |
| Recreation | 3.27 | 0.70 | 0.36 | 0.03 | 0.22 | 0.06 |
| Culture | 0.00 | 0.00 | 0.32 | 0.00 | 0.06 |  |
| **Total** | **3.80** | **4.36** | **4.13** | **0.16** | **13.41** | **4.11** |

Appendix 5: Ecosystem service value for different LULC for the year of 2027

| **Ecosystem Services** | **Ecosystem Service value (US Million$/ha./year) for 2027** | | | | | |
| --- | --- | --- | --- | --- | --- | --- |
|  | **Built-up** | **Vegetation** | **Marshy land** | **River** | **Wetland** | **DAF land** |
| **Provisional services** |  |  |  |  |  |  |
| Water supply | 0.00 | 0.03 | 0.06 | 0.02 | 0.08 | 0.22 |
| Food production | 0.00 | 0.21 | 0.10 | 0.00 | 0.08 | 1.25 |
| Raw materials | 0.00 | 0.09 | 0.09 | 0.00 | 0.04 | 0.12 |
| Genetic resources | 0.00 | 1.56 | 0.02 | 0.00 | 0.02 | 0.56 |
| **Regulating services** |  |  |  |  |  |  |
| Water regulation | 0.01 | 0.01 | 0.89 | 0.10 | 0.15 | 0.00 |
| Waste treatment | 0.00 | 0.12 | 0.48 | 0.01 | 9.61 | 0.21 |
| Erosion control | 0.00 | 0.35 | 0.41 | 0.00 | 0.30 | 0.06 |
| Climate regulation | 0.53 | 2.11 | 0.08 | 0.00 | 0.02 | 0.22 |
| Biological control | 0.00 | 0.01 | 0.15 | 0.00 | 0.03 | 0.02 |
| Gas regulation | 0.00 | 0.01 | 0.00 | 0.00 | 0.00 | 0.00 |
| Disturbance regulation | 0.00 | 0.07 | 0.47 | 0.00 | 0.40 | 0.00 |
| **Supporting services** |  |  |  |  |  |  |
| Nutrient cycling | 0.00 | 0.00 | 0.27 | 0.00 | 0.05 | 0.00 |
| Pollution | 0.00 | 0.03 | 0.00 | 0.00 | 0.00 | 0.01 |
| Soil formation | 0.00 | 0.01 | 0.00 | 0.00 | 0.00 | 0.29 |
| Habitat/Refugio | 0.00 | 0.04 | 0.39 | 0.00 | 1.07 | 0.00 |
| **Cultural services** |  |  |  |  |  |  |
| Recreation | 3.37 | 0.89 | 0.35 | 0.03 | 0.19 | 0.04 |
| Culture | 0.00 | 0.00 | 0.32 | 0.00 | 0.05 | 0.00 |
| **Total** | **3.90** | **5.55** | **4.07** | **0.17** | **12.10** | **3.01** |

Appendix 6: Ecosystem service value for different LULC for the year of 2036

| **Ecosystem Services** | **Ecosystem Service value (US Million$/ha./year) for 2036** | | | | | |
| --- | --- | --- | --- | --- | --- | --- |
|  | **Built-up** | **Vegetation** | **Marshy land** | **River** | **Wetland** | **DAF land** |
| **Provisional services** |  |  |  |  |  |  |
| Water supply | 0.00 | 0.03 | 0.06 | 0.02 | 0.07 | 0.24 |
| Food production | 0.00 | 0.20 | 0.08 | 0.00 | 0.07 | 1.37 |
| Raw materials | 0.00 | 0.08 | 0.07 | 0.00 | 0.03 | 0.13 |
| Genetic resources | 0.00 | 1.52 | 0.01 | 0.00 | 0.02 | 0.61 |
| **Regulating services** |  |  |  |  |  |  |
| Water regulation | 0.01 | 0.01 | 0.08 | 0.10 | 0.13 | 0.00 |
| Waste treatment | 0.00 | 0.12 | 0.42 | 0.01 | 7.80 | 0.23 |
| Erosion control | 0.00 | 0.34 | 0.36 | 0.00 | 0.25 | 0.06 |
| Climate regulation | 0.55 | 2.04 | 0.07 | 0.00 | 0.01 | 0.24 |
| Biological control | 0.00 | 0.01 | 0.13 | 0.00 | 0.02 | 0.02 |
| Gas regulation | 0.00 | 0.01 | 0 | 0.00 | 0.00 | 0.00 |
| Disturbance regulation | 0.00 | 0.07 | 0.41 | 0.00 | 0.32 | 0.00 |
| **Supporting services** |  |  |  |  |  |  |
| Nutrient cycling | 0.00 | 0.00 | 0.24 | 0.00 | 0.04 | 0.00 |
| Pollution | 0.00 | 0.03 | 0 | 0.00 | 0.00 | 0.01 |
| Soil formation | 0.00 | 0.01 | 0 | 0.00 | 0.00 | 0.31 |
| Habitat/Refugio | 0.00 | 0.04 | 0.34 | 0.00 | 0.87 | 0.00 |
| **Cultural services** |  |  | 0 |  |  |  |
| Recreation | 3.47 | 0.87 | 0.31 | 0.03 | 0.15 | 0.05 |
| Culture | 0.00 | 0.00 | 0.28 | 0.00 | 0.04 | 0.00 |
| **Total** | **4.03** | **5.38** | **3.55** | **0.16** | **9.81** | **3.27** |

Appendix 7: Ecosystem service value for different LULC for the year of 2045

| **Ecosystem Services** | **Ecosystem Service value (US Million$/ha./year) for 2045** | | | | | |
| --- | --- | --- | --- | --- | --- | --- |
|  | **Built-up** | **Vegetation** | **Marshy land** | **River** | **Wetland** | **DAF land** |
| **Provisional services** |  |  |  |  |  |  |
| Water supply | 0.00 | 0.03 | 0.05 | 0.02 | 0.03 | 0.25 |
| Food production | 0.00 | 0.20 | 0.07 | 0.00 | 0.03 | 1.46 |
| Raw materials | 0.00 | 0.08 | 0.06 | 0.00 | 0.01 | 0.14 |
| Genetic resources | 0.00 | 1.49 | 0.01 | 0.00 | 0.01 | 0.65 |
| **Regulating services** |  |  |  |  |  |  |
| Water regulation | 0.01 | 0.01 | 0.66 | 0.10 | 0.05 | 0.00 |
| Waste treatment | 0.00 | 0.12 | 0.36 | 0.01 | 2.98 | 0.25 |
| Erosion control | 0.00 | 0.33 | 0.31 | 0.00 | 0.09 | 0.07 |
| Climate regulation | 0.56 | 2.00 | 0.06 | 0.00 | 0.01 | 0.26 |
| Biological control | 0.00 | 0.01 | 0.11 | 0.00 | 0.01 | 0.02 |
| Gas regulation | 0.00 | 0.01 | 0.00 | 0.00 | 0.00 | 0.00 |
| Disturbance regulation | 0.00 | 0.06 | 0.35 | 0.00 | 0.12 | 0.00 |
| **Supporting services** |  |  |  |  |  |  |
| Nutrient cycling | 0.00 | 0.00 | 0.20 | 0.00 | 0.02 | 0.00 |
| Pollution | 0.00 | 0.03 | 0.00 | 0.00 | 0.00 | 0.01 |
| Soil formation | 0.00 | 0.01 | 0.00 | 0.00 | 0.00 | 0.33 |
| Habitat/Refugio | 0.00 | 0.04 | 0.29 | 0.00 | 0.33 | 0.00 |
| **Cultural services** |  |  |  |  |  |  |
| Recreation | 3.57 | 0.85 | 0.26 | 0.03 | 0.06 | 0.05 |
| Culture | 0.00 | 0.00 | 0.24 | 0.00 | 0.02 | 0.00 |
| **Total** | **4.14** | **5.28** | **3.04** | **0.17** | **3.74** | **3.49** |

Appendix 8: Ecosystem service value for different LULC for the year of 2027 based on proposed plan

| **Ecosystem Services** | **2027** | | | | | |
| --- | --- | --- | --- | --- | --- | --- |
|  | **Built-up** | **Vegetation** | **Marshy land** | **River** | **Wetland** | **DAF land** |
| **Provisional services** |  |  |  |  |  |  |
| Water supply | 0.00 | 0.03 | 0.07 | 0.02 | 0.10 | 0.19 |
| Food production | 0.00 | 0.21 | 0.11 | 0.00 | 0.10 | 1.11 |
| Raw materials | 0.00 | 0.09 | 0.10 | 0.00 | 0.04 | 0.10 |
| Genetic resources | 0.00 | 1.56 | 0.02 | 0.00 | 0.02 | 0.50 |
| **Regulating services** |  |  |  |  |  |  |
| Water regulation | 0.01 | 0.01 | 1.00 | 0.10 | 0.18 | 0.00 |
| Waste treatment | 0.00 | 0.12 | 0.54 | 0.01 | 11.42 | 0.19 |
| Erosion control | 0.00 | 0.35 | 0.47 | 0.00 | 0.36 | 0.05 |
| Climate regulation | 0.56 | 2.11 | 0.09 | 0.00 | 0.02 | 0.20 |
| Biological control | 0.00 | 0.01 | 0.17 | 0.00 | 0.03 | 0.02 |
| Gas regulation | 0.00 | 0.01 | 0.00 | 0.00 | 0.00 | 0.00 |
| Disturbance regulation | 0.00 | 0.07 | 0.53 | 0.00 | 0.47 | 0.00 |
| **Supporting services** |  |  |  |  |  |  |
| Nutrient cycling | 0.00 | 0.00 | 0.31 | 0.00 | 0.06 | 0.00 |
| Pollution | 0.00 | 0.03 | 0.00 | 0.00 | 0.00 | 0.01 |
| Soil formation | 0.00 | 0.01 | 0.00 | 0.00 | 0.00 | 0.25 |
| Habitat/Refugia | 0.00 | 0.04 | 0.44 | 0.00 | 1.28 | 0.00 |
| **Cultural services** |  |  |  |  |  |  |
| Recreation | 3.52 | 0.89 | 0.39 | 0.03 | 0.23 | 0.04 |
| Culture | 0.00 | 0.00 | 0.36 | 0.00 | 0.07 | 0.00 |
| **Total** | **4.09** | **5.55** | **4.58** | **0.17** | **14.38** | **2.65** |

Appendix 9: Ecosystem service value for different LULC for the year of 2036 based on proposed plan

| **Ecosystem Services** | **2036** | | | | | |
| --- | --- | --- | --- | --- | --- | --- |
|  | **Built-up** | **Vegetation** | **Marshy land** | **River** | **Wetland** | **DAF land** |
| **Provisional services** |  |  |  |  |  |  |
| Water supply | 0.00 | 0.03 | 0.07 | 0.02 | 0.10 | 0.17 |
| Food production | 0.00 | 0.21 | 0.11 | 0.00 | 0.10 | 1.00 |
| Raw materials | 0.00 | 0.09 | 0.10 | 0.00 | 0.04 | 0.09 |
| Genetic resources | 0.00 | 1.56 | 0.02 | 0.00 | 0.02 | 0.45 |
| **Regulating services** |  |  |  |  |  |  |
| Water regulation | 0.01 | 0.01 | 1.00 | 0.10 | 0.18 | 0.00 |
| Waste treatment | 0.00 | 0.12 | 0.54 | 0.01 | 11.41 | 0.17 |
| Erosion control | 0.00 | 0.35 | 0.46 | 0.00 | 0.36 | 0.05 |
| Climate regulation | 0.60 | 2.11 | 0.09 | 0.00 | 0.02 | 0.18 |
| Biological control | 0.00 | 0.01 | 0.17 | 0.00 | 0.03 | 0.01 |
| Gas regulation | 0.00 | 0.01 | 0.00 | 0.00 | 0.00 | 0.00 |
| Disturbance regulation | 0.00 | 0.07 | 0.53 | 0.00 | 0.47 | 0.00 |
| **Supporting services** |  |  |  |  |  |  |
| Nutrient cycling | 0.00 | 0.00 | 0.31 | 0.00 | 0.06 | 0.00 |
| Pollution | 0.00 | 0.03 | 0.00 | 0.00 | 0.00 | 0.01 |
| Soil formation | 0.00 | 0.01 | 0.00 | 0.00 | 0.00 | 0.23 |
| Habitat/Refugia | 0.00 | 0.04 | 0.44 | 0.00 | 1.28 | 0.00 |
| **Cultural services** |  |  |  |  |  |  |
| Recreation | 3.78 | 0.89 | 0.39 | 0.03 | 0.23 | 0.04 |
| Culture | 0.00 | 0.00 | 0.36 | 0.00 | 0.07 | 0.00 |
| **Total** | **4.39** | **5.54** | **4.58** | **0.16** | **14.37** | **2.40** |

Appendix 10: Ecosystem service value for different LULC for the year of 2045 based on proposed plan

| **Ecosystem Services** | **2045** | | | | | |
| --- | --- | --- | --- | --- | --- | --- |
|  | **Built-up** | **Vegetation** | **Marshy land** | **River** | **Wetland** | **DAF land** |
| **Provisional services** |  |  |  |  |  |  |
| Water supply | 0.00 | 0.03 | 0.07 | 0.02 | 0.10 | 0.15 |
| Food production | 0.00 | 0.21 | 0.11 | 0.00 | 0.10 | 0.90 |
| Raw materials | 0.00 | 0.09 | 0.10 | 0.00 | 0.04 | 0.08 |
| Genetic resources | 0.00 | 1.56 | 0.02 | 0.00 | 0.02 | 0.40 |
| **Regulating services** |  |  |  |  |  |  |
| Water regulation | 0.01 | 0.01 | 1.00 | 0.10 | 0.18 | 0.00 |
| Waste treatment | 0.00 | 0.12 | 0.54 | 0.01 | 11.40 | 0.15 |
| Erosion control | 0.00 | 0.35 | 0.46 | 0.00 | 0.36 | 0.04 |
| Climate regulation | 0.64 | 2.11 | 0.09 | 0.00 | 0.02 | 0.16 |
| Biological control | 0.00 | 0.01 | 0.17 | 0.00 | 0.03 | 0.01 |
| Gas regulation | 0.00 | 0.01 | 0.00 | 0.00 | 0.00 | 0.00 |
| Disturbance regulation | 0.00 | 0.07 | 0.53 | 0.00 | 0.47 | 0.00 |
| **Supporting services** |  |  |  |  |  |  |
| Nutrient cycling | 0.00 | 0.00 | 0.31 | 0.00 | 0.06 | 0.00 |
| Pollution | 0.00 | 0.03 | 0.00 | 0.00 | 0.00 | 0.01 |
| Soil formation | 0.00 | 0.01 | 0.00 | 0.00 | 0.00 | 0.21 |
| Habitat/Refugia | 0.00 | 0.04 | 0.44 | 0.00 | 1.28 | 0.00 |
| **Cultural services** |  |  |  |  |  |  |
| Recreation | 4.04 | 0.89 | 0.39 | 0.03 | 0.23 | 0.03 |
| Culture | 0.00 | 0.00 | 0.36 | 0.00 | 0.07 | 0.00 |
| **Total** | **4.69** | **5.55** | **4.58** | **0.17** | **14.36** | **2.15** |

**Description of the Landsat images used for this study**

| **Landsat Type** | **Path/ Row** | **Date of Acquisition** | **Band Combination Used for LULC** | **Radiance values of the selected bands** | | **Reflectance values of the selected bands** | | **Pixel values of the selected bands** | |
| --- | --- | --- | --- | --- | --- | --- | --- | --- | --- |
|  |  |  |  | ***Maximum*** | ***Minimum*** | ***Maximum*** | ***Minimum*** | ***Maximum*** | ***Minimum*** |
| TM | 139/ 43 | 8/11/1991 | (BAND 4+BAND 3+BAND 2) | BAND 2 = 333.000 | BAND 2 = -2.840 | BAND 2 = 0.583892 | BAND 2 = -0.004980 | BAND 2 = 51 | BAND 2 = 30 |
|  |  |  |  | BAND 3 = 264.000 | BAND 3 = -1.170 | BAND 3 = 0.546477 | BAND_3 = -0.002422 | BAND 3 = 62 | BAND 3 = 26 |
|  |  |  |  | BAND 4 = 221.000 | BAND 4 = -1.510 | BAND 4 = 0.659851 | BAND 4 = -0.004508 | BAND 4 = 86 | BAND 4 = 24 |
| TM | 139/ 43 | 16/11/2000 | (BAND 4+BAND 3+BAND 2) | BAND 2 = 365.000 | BAND 2 = -2.840 | BAND 2 = 0.637433 | BAND 2 = -0.004960 | BAND 2 = 63 | BAND 2 = 25 |
|  |  |  |  | BAND 3 = 264.000 | BAND 3 = -1.170 | BAND 3 = 0.544284 | BAND 3 = -0.002412 | BAND 3 = 76 | BAND 3 = 22 |
|  |  |  |  | BAND 4 = 221.000 | BAND 4 = -1.510 | BAND 4 = 0.657203 | BAND 4 = -0.004490 | BAND 4 = 89 | BAND 4 = 18 |
| TM | 139/ 43 | 9/11/2009 | (BAND 4+BAND 3+BAND 2) | BAND 2 = 365.000 | BAND 2 = -2.840 | BAND 2 = 0.639594 | BAND 2 = -0.004977 | BAND 2 = 57 | BAND 2 = 28 |
|  |  |  |  | BAND 3 = 264.000 | BAND 3 = -1.170 | BAND 3 = 0.546129 | BAND 3 = -0.002420 | BAND 3 = 81 | BAND 3 = 23 |
|  |  |  |  | BAND 4 = 221.000 | BAND 4 = -1.510 | BAND 4 = 0.659431 | BAND 4 = -0.004506 | BAND 4 = 111 | BAND 4 = 20 |
| OLI | 139/ 43 | 18/11/2018 | (BAND 5+BAND 4+BAND 3) | BAND 3 = 733.94958 | BAND 3 = -60.60979 | BAND 3 = 1.210700 | BAND 3 = -0.099980 | BAND 3 = 11998 | BAND 3 = 8242 |
|  |  |  |  | BAND 4 = 618.90759 | BAND 4 = -51.10959 | BAND 4 = 1.210700 | BAND 4 = -0.099980 | BAND 4 = 12328 | BAND 4 = 7399 |
|  |  |  |  | BAND 5 = 378.74060 | BAND 5 = -31.27652 | BAND 5 = 1.210700 | BAND 5 = -0.099980 | BAND 5 = 19777 | BAND 5 = 7036 |
